# Nurse Telephone Support for Caregivers of Older Adults at Hospital Discharge: A Randomized Clinical Trial

**DOI:** 10.1001/jamanetworkopen.2024.41019

**Published:** 2024-10-25

**Authors:** Anne-Marie Hill, Wendy Moyle, Susan Slatyer, Christina Bryant, Keith D. Hill, Nicholas Waldron, Samar Aoun, Ami Kamdar, Laurie Grealish, Caroline Reberger, Cindy Jones, Mary Bronson, Max K. Bulsara, Angela Jacques, Cheng Yen Loo, Sean Maher

**Affiliations:** 1 School of Allied Health, University of Western Australia, Perth, Western Australia, Australia; 2 Western Australia Centre for Health and Ageing, University of Western Australia, Perth, Western Australia, Australia; 3 Menzies Health Institute Queensland, Griffith University, Brisbane, Queensland, Australia; 4 School of Nursing and Midwifery, Griffith University, Brisbane, Queensland, Australia; 5 School of Nursing, Centre for Healthy Ageing, Murdoch University, Perth, Western Australia, Australia; 6 Centre for Nursing Research, Sir Charles Gairdner Osborne Park Health Care Group, Nedlands, Western Australia, Australia; 7 Melbourne School of Psychological Sciences, University of Melbourne, Melbourne, Victoria, Australia; 8 Rehabilitation, Ageing and Independent Living Research Centre, Monash University, Peninsula Campus, Victoria, Australia; 9 Department of Rehabilitation and Aged Care, Armadale Health Service, Perth, Western Australia, Australia; 10 Medical School, University of Western Australia, Perth, Western Australia, Australia; 11 Perron Institute, Perth, Western Australia, Australia; 12 La Trobe University, Melbourne, Victoria, Australia; 13 Department of General Medicine, Sir Charles Gairdner Hospital, Nedlands, Western Australia, Australia; 14 Menzies Health Institute Queensland, Griffith University, Gold Coast, Queensland, Australia; 15 Queensland Nursing and Midwifery Education and Research Unit, Gold Coast Hospital and Health Service, Gold Coast, Queensland, Australia; 16 Social Work Department, Sir Charles Gairdner Hospital, Nedlands, Western Australia, Australia; 17 Faculty of Health Sciences and Medicine, Bond University, Gold Coast, Queensland, Australia; 18 Specialty and Ambulatory Services, Sir Charles Gairdner Osborne Park Health Care Group, Perth, Western Australia, Australia; 19 Institute for Health Research, University of Notre Dame Australia, Fremantle, Western Australia, Australia; 20 Department of Geriatric, Acute and Rehabilitation Medicine, Sir Charles Gairdner Hospital, Nedlands, Western Australia, Australia

## Abstract

**Importance:**

Informal caregivers who provide home-based care frequently experience stress and burden that adversely affect their health-related quality of life (HRQOL).

**Objective:**

To evaluate the efficacy of the Further Enabling Care at Home (FECH^+^) program for the HRQOL of caregivers of older adults discharged home from the hospital.

**Design, Setting, and Participants:**

This multicenter, parallel, 2-group randomized clinical trial, with blinded baseline and outcome measurements, was conducted at 3 hospitals in 2 states in Australia. Recruitment took place between August 2020 and July 2022, and follow-up was performed for 12 months after hospital discharge. Participants were dyads of caregivers and patients. Eligible caregivers were aged 18 years or older who provided informal home-based care at least weekly for a patient aged 70 years or older. Caregivers were enrolled when their patient was discharged from the hospital. Dyads were randomly assigned to either the intervention or control group. Data analysis followed an intention-to-treat approach.

**Intervention:**

Caregivers in the intervention group received the FECH^+^ program, structured nurse support of 6 telephone calls over 6 months after the patient’s discharge plus usual discharge care. Caregivers in the control group received usual care alone.

**Main Outcomes and Measures:**

Primary outcome was caregivers’ HRQOL 6 months after discharge, which was measured using the Assessment of Quality of Life 8-Dimension (AQOL-8D). Secondary outcomes were caregivers’ HRQOL 12 months after discharge as well as preparedness to care (measured using the Preparedness for Caregiving Scale), self-efficacy (measured using the Caregiver Inventory), and levels of strain and distress (measured using the Family Appraisal of Caregiving Questionnaire) at 6 and 12 months after discharge. Baseline and outcome measurements were administered by telephone at 3, 6, and 12 months after discharge.

**Results:**

A total of 547 dyads (caregivers: 405 females [74.0%], mean [SD] age, 64.50 [12.82] years; patients: 296 females [54.1%], mean [SD] age, 83.16 [7.04] years for the intervention group and 83.45 [7.20] years for the control group) were included in the intention-to-treat analysis. There was no significant difference in caregivers’ HRQOL between the 2 groups at the primary time point of 6 months (difference in AQOL-8D score, 0.01; 95% CI, −0.02 to 0.03; *P* = .62) after hospital discharge.

**Conclusions and Relevance:**

In this randomized clinical trial, the FECH^+^ program—a nurse telephone support intervention for caregivers of older adults after hospital discharge—did not significantly improve caregivers’ HRQOL at 6 months after discharge compared with usual care. Additional examination is warranted into improving caregivers’ HRQOL at the time of their patient’s hospital discharge.

**Trial Registration:**

Australian New Zealand Clinical Trials Registry Identifier: ACTRN12620000060943

## Key Points

**Question:**

Can a nurse telephone support intervention improve health-related quality of life (HRQOL) for caregivers of older adults after hospital discharge?

**Findings:**

In this randomized clinical trial of 547 caregiver and patient dyads, caregivers who received 6 supportive telephone calls from a nurse over 6 months after the patient’s discharge from the hospital experienced no significant improvement in HRQOL compared with caregivers who received usual discharge care alone.

**Meaning:**

Findings of this study suggest that further research is needed into how to improve caregivers’ HRQOL when older patients are discharged from the hospital.

## Teaser

This randomized clinical trial evaluates whether a telephone-based service, the Further Enabling Care at Home+ program, which supports home-based informal caregivers after hospital discharge of their older care recipients, improves caregivers’ health-related quality of life.

## Introduction

Approximately 11% to 20% of the population in Australia, the US, and the UK provide informal, unpaid care, including physical, social, and psychological support for family and friends.^1,2,3^ Annually, this informal care is estimated to be worth £162 million in the UK and $600 billion in the US.^2,4^ Aging of the global population raises the importance of informal care provision and the urgent need to support informal caregivers of older adults.^1,5,6^ The proportion of adults aged 65 years or older, both in the US and Australia, will increase from 16% in 2022 to approximately 23% by 2050,^7,8^ consistent with the global trajectory.^9^ Informal caregiving can be rewarding but physically, psychosocially, and financially challenging^1,3,10,11,12^ and is associated with high levels of stress, poor health, and reduced health-related quality of life (HRQOL).^13,14,15,16,17^

These caregivers have reported feeling unprepared for caregiving; being overwhelmed and distressed; and facing substantial gaps in health care and services, including no support or acknowledgment from health professionals, when the older adult they care for is discharged from the hospital.^2,18,19,20^ These gaps in preparedness to care may lead to serious problems for the caregiver and the patient because older adults discharged home from the hospital are at risk for functional decline, adverse events, and unplanned readmissions to the hospital.^21,22,23^ Review articles have identified that supported discharge for older adults may be associated with reduced hospital readmissions, but its role in improved patient health or caregiver outcomes is uncertain.^24,25,26,27^ Educational and psychosocial interventions intended to support caregivers of older persons after discharge have not been found effective in improving HRQOL or preparedness to care.^28,29^ Studies have focused on limited populations of patients, mainly those with dementia and stroke, and reviews of studies of caregiver interventions rate the risk of bias as high.^29,30,31^ Therefore, high-quality evidence is required on how to support caregivers’ preparedness to care after discharge and address the impact of caregiving on caregiver health and well-being.^31^

To address this gap, a trial for the Further Enabling Care at Home (FECH^+^) program, a nurse support telephone intervention for caregivers of older adults discharged home from the hospital, was conducted.^32,33^ The primary aim of the trial was to evaluate the efficacy of the FECH^+^ program for the HRQOL of caregivers of older adults at 6 months after discharge from the hospital. Secondary aims included evaluating the efficacy of the FECH^+^ program for caregivers’ (1) HRQOL 12 months after discharge as well as (2) preparedness to care, (3) self-efficacy, and (4) levels of strain and distress at 6 and 12 months after discharge.

## Methods

### Design

This single-blind, multicenter, parallel, 2-group randomized clinical trial, with a 1:1 dyad randomization to groups, was conducted in Australia. The trial protocol (Supplement 1) is published elsewhere.^33^ Dyads of caregiver and patient were enrolled between August 2020 and July 2022. A consumer representative provided advice to the researchers about procedures throughout the trial.^33^ Economic and process evaluations, including evaluation of patient outcomes, will be reported separately.^33^ The Sir Charles Gairdner and Osborne Park Health Care Group, Department of Health Western Australia, The University of Western Australia, Griffith University, and Gold Coast Hospital and Health Service Ethics Committees approved the trial. All participants provided written informed consent. Patients who were unable to provide informed consent due to cognitive impairment were recruited if their caregiver was willing to participate in the trial and a waiver of consent or proxy consent was obtained from their legal guardian. We followed the Consolidated Standards of Reporting Trials (CONSORT) reporting guideline.^34^

### Setting and Participants

The trial was conducted at 3 hospitals in 2 states (Western Australia and Queensland) in Australia. The setting in Western Australia is a 600-bed tertiary teaching hospital in Perth that serves statewide adult populations. Included wards admit adults who are predominantly aged 65 years or older for acute medical treatment, including a short-stay medical assessment unit that predominantly admits this population. In Queensland, 2 Gold Coast hospitals enrolled patients from medical wards. One site is a 600-bed tertiary teaching hospital, and the other site is a 400-bed teaching hospital with services that include emergency, general medical, and surgical care and general rehabilitation.

Participants were recruited as dyads: caregiver and patient. Eligibility criteria for caregivers were aged 18 years or older and providing unpaid support on an ongoing basis (at least weekly) to a person aged 70 years or older discharged home from a participating hospital ward. Care was required to be home based and could be physical, social, and/or emotional.^1,35^ Patients with any medical diagnosis were eligible if they were discharged home from a participating hospital ward and were aged 70 years or older. Dyads were excluded from the FECH^+^ program if the patient was not discharged directly to the home from a medical ward (eg, discharged to a residential care facility or transferred to a surgical ward) and if the caregiver could not communicate proficiently in English or their hearing prohibited holding a telephone conversation.

### Randomization and Blinding

The Western Australia Health Translation Network’s Clinical Trial and Data Management Centre (CTDMC) independently managed the randomization procedure. The CTDMC is managed by Western Australia’s state-based research translation center, which supports state researchers to conduct high-quality clinical trials. The treatment allocation list was generated by the CTDMC using computer-generated random numbers and was accessible only to the CTDMC administrators.

Trial staff who recruited participants and completed baseline and follow-up measurements were rigorously blinded to group allocation through the externally controlled online randomization procedure and independently managed the telephone follow-up assessments. Staff who conducted recruitment and outcome measurement worked in a separate location from the FECH^+^ program nurses who were responsible for delivering the intervention. All measurement data were entered electronically, and online participant registration by the baseline assessor triggered automatic allocation to a group. Dyad allocation to the intervention group triggered an electronic communication to nurses who commenced delivering the intervention. Nurses entered each intervention occasion of service electronically and knew which participant received the intervention, but they had no contact with the control group dyads and were not involved in the baseline or follow-up data collection.

Hospital staff, including those who organized discharge services, were blinded to dyads’ enrollment. Participants were not informed of their group allocation but could not be blinded to the intervention they received. Participants were instructed not to disclose their treatment allocation to trial staff during follow-up assessments.

### Intervention and Control Conditions

Caregivers in the intervention group received the FECH^+^ program plus usual discharge care; the FECH^+^ program is summarized using a Template for Intervention Description and Replication (eTable 1 in Supplement 2).^36^ The FECH^+^ program was a nurse-led postdischarge intervention delivered via 6 telephone calls. Caregivers were contacted by telephone in the first and second week after discharge and subsequently at 1, 2, 4, and 6 months after discharge. The theoretical basis for the intervention was Problem Solving Therapy^37^ using the ADAPT (Attitude, Define, Alternatives, Predict and Try Out) approach.^38^ During the first call, the nurse focused on eliciting the caregiver’s understanding of the hospital discharge information. Key components of subsequent calls were problem orientation, definition and formulation, alternatives generation, decision-making, and solution implementation. The CSNAT (Carer Support Needs Assessment Tool^39^) was used by nurses to guide participants through the initial 2 stages and enabled caregivers to identify and prioritize up to 3 areas where they needed support.^39,40^ After each call, all participants were sent by mail or email a program booklet and follow-up resources, as appropriate.

All FECH^+^ program nurses had gerontological expertise and proficiency in navigating their state home care system. Tailored telephone calls of necessity differed between each nurse and caregiver. Fidelity was addressed by nurses receiving 3 days of training, using the CSNAT, completing an online checklist at each contact, and attending regular online meetings during the trial.

Dyads in both control and intervention groups received usual discharge care. Usual care entailed receiving a copy of the discharge letter provided to the patient’s general practitioner, medications, a prescription list with instructions, and outpatient appointments if required.

### Outcomes and Measures

Primary and secondary outcomes were measured at baseline, 3, 6, and 12 months after discharge. The primary outcome was the caregiver’s HRQOL at 6 months after the patient’s discharge from the hospital measured using the Assessment of Quality of Life 8-Dimension (AQOL-8D). The AQOL-8D is a multiattribute utility instrument consisting of 35 questions that measure 8 dimensions (5 psychosocial and 3 physical) of an individual’s health and a score range of 0 to 1, with higher scores indicating better HRQOL (eFigure 1 in Supplement 2).^41,42^ Secondary outcomes (described in the eMethods in Supplement 2) were the caregiver’s (1) HRQOL at 12 months after discharge; (2) preparedness to care at 6 and 12 months after discharge measured with the Preparedness for Caregiving Scale (PCS), an 8-item questionnaire that uses a Likert scale (0 for not at all prepared to 4 for very well prepared) to rate a caregiver’s perception of their readiness to provide support to the patient^43,44^ and is scored by calculating the mean of all items answered (score range: 0-4, with higher scores indicating greater preparedness for caregiving); (3) self-efficacy at 6 and 12 months after discharge measured using the Caregiver Inventory (CGI), a reliable and validated 21-item questionnaire that uses a Likert scale (1 for not at all confident to 9 for totally confident) to assess caregiving self-efficacy expectations^45,46^ (score range: 21-189, with higher scores indicating better self-efficacy); and (4) levels of strain and distress at 6 and 12 months after discharge measured with the Family Appraisal of Caregiving Questionnaire (FACQ) using 2 (an 8-item and a 5-item) subscales; each item is scored using a 5-point Likert scale (1 for strongly agree to 5 for strongly disagree; total score range: 5-25 [levels of strain]; 8-30 [levels of distress], with higher scores indicating lower levels of strain and distress).^47^

Caregiver demographic data gathered at baseline included age, sex, language, educational level, number and type of health conditions, number of medications, and amount of assistance provided to the patient. Patient demographic data collected included age, sex, length of stay in hospital, functional ability (measured using the Barthel Index for Activities of Daily Living; score range: 0-100, with higher scores indicating greater independence in undertaking activities of daily living^48^), and distress from symptoms of illness (measured using the Symptom Assessment Scale; score range: 0-70, with higher scores indicating higher levels of symptom distress^49^).

### Sample Size

The trial was designed with 80% power (α<.05) to detect an effect size of 0.22 in the primary outcome (change in utility score on the AQOL-8D instrument) for the caregiver at 6 months after discharge. This effect size was based on findings from the earlier pilot trial of a similar intervention.^32,33^ The sample size required was 648 dyads (324 in each group), determined using the G*Power sample size calculator.^50^ Based on preliminary work, this sample size was also expected to have 80% power to detect meaningful differences in the secondary outcomes.^33^ The trial aimed to enroll 925 dyads to allow for a 30% dropout rate due to the older population being recruited.

### Protocol Deviation

The COVID-19 pandemic did not affect trial procedures in the Western Australian site. However, it was estimated that original recruitment targets would not be achieved at the Queensland sites due to pandemic restrictions. Recalculation of the power indicated that a sample size of 506 dyads (253 in each group) would have 80% power to detect an effect size of 0.25. A revised dropout rate of approximately 10% (based on interim recruitment data, which indicated high levels of dyad retention in Western Australia) meant that 551 dyads were required. To allow for uncertainty around pandemic restrictions in Western Australia, recruitment continued with the aim to recruit 588 dyads (allowing for a 13% dropout rate). The final attrition rate (7.5%) meant that the number of participants required to detect an effect was achieved according to the revised calculation.

Recruitment of participants at the Queensland sites commenced in February 2021 and ceased in December 2021. Pandemic restrictions prevented researchers from entering hospital wards and undertaking screening or recruitment for 3 months. Additional intermittent restrictions caused interruptions to screening and recruitment and altered admission patterns to participating wards.

### Statistical Analysis

Analyses were completed using Stata, version 17.1 (StataCorp LLC) and an intention-to-treat approach.^33^ Participant demographic and other characteristics were recoded into categorical variables when appropriate. Demographic data were summarized using frequency distributions for categorical data and means (SDs) or medians (IQRs and ranges) for continuous data. χ^2^ Tests and unpaired, 2-tailed *t* tests or Mann-Whitney tests, as appropriate, were used for categorical and continuous data comparisons between dyads in the intervention and control groups. All outcome data were summarized using similar descriptive statistics.

Generalized linear mixed-effects models with random effects were used to examine and describe the longitudinal primary outcome (HRQOL at 6 months) and secondary outcomes (HRQOL at 12 months and PCS, CGI, and FACQ scores at 6 and 12 months) over 4 time points (baseline, 3, 6, and 12 months). Outcome data distributions were assessed for appropriate transformation or model selections graphically and using ladder of powers, a command that searches a table of transformations for one that converts the outcome variable into a normally distributed variable.^51^ Models included linear mixed models with transformed and raw data, mixed-effects tobit models, and mixed-effects γ models depending on data distribution. Model results were summarized using marginal mean and mean difference estimates with 95% CIs. All models were adjusted for baseline covariates that are known to be associated with the outcomes (caregiver sex, multicomorbidity, polypharmacy, educational level, employment status, caregiver role training, needing to care for other people, and patient Barthel Index for Activities of Daily Living and Symptom Assessment Scale scores). Given that mixed-effects models use maximum likelihood estimation to estimate parameters based on assumed probability distributions, all available data points, regardless of missing time points, were included in analyses. All statistical tests were 2-sided, and statistical significance was determined at *P* < .05.

## Results

**Figure 1.**
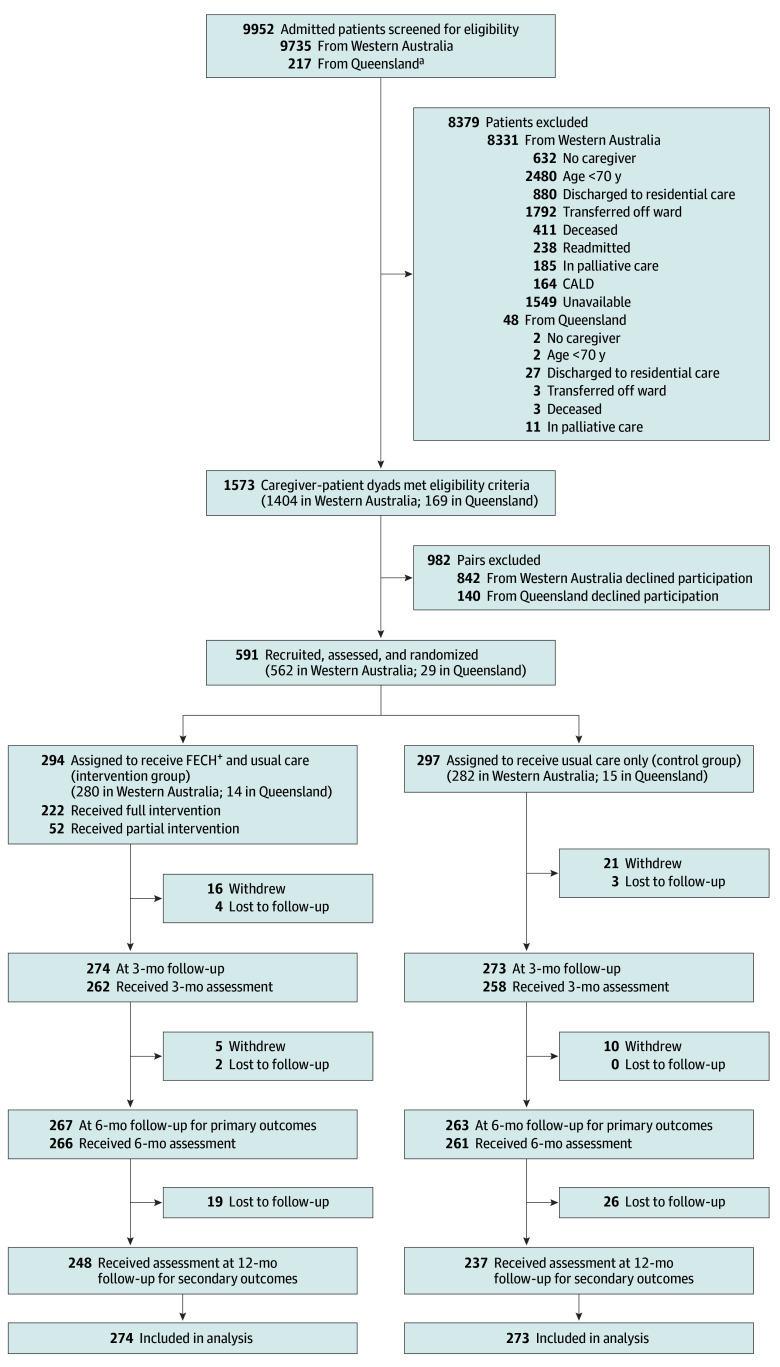
Participant Flow Through the Trial CALD indicates culturally and linguistically diverse; FECH^+^, Further Enabling Care at Home. ^a^Screening in Queensland was interrupted due to COVID-19 pandemic–related ward access restrictions.

Figure 1 presents participants’ flow through the trial. Although 591 dyads were recruited, screened, and randomized, 44 withdrew or were lost to follow-up before the first follow-up measurement at 3 months. A total of 547 dyads (274 in the intervention group, 273 in the control group) were included in the intention-to-treat analysis.

Participant characteristics are presented in Table 1. Caregivers (405 females [74.0%], 142 males [26.0%]; mean [SD] age, 64.50 [12.82] years) were more frequently daughters of the patients and older adults. Patients (296 females [54.1%], 251 males [45.9%]; mean [SD] age, 83.16 [7.04] years for the intervention group, 83.45 [7.20] years for the control group) most frequently lived with their caregiver (intervention group: 62.8% [172]; control group: 64.1% [175]).

**Table 1.** Caregiver and Patient Characteristics

Characteristic	Participants, No. (%)	
	Intervention group (n = 274)	Control group (n = 273)
**Caregivers**		
Age, mean (SD), y	64.02 (12.48)	65.05 (13.16)
Sex		
Male	68 (24.8)	74 (27.1)
Female	206 (75.2)	199 (72.9)
Birthplace		
Australia	175 (63.9)	187 (68.5)
UK	37 (13.5)	34 (12.5)
New Zealand	10 (3.7)	2 (0.7)
Italy	5 (1.8)	7 (2.6)
India	4 (1.5)	5 (1.8)
South Africa	4 (1.5)	3 (1.1)
Malaysia	3 (1.1)	4 (1.5)
Other^a^	36 (13.1)	31 (11.4)
Relationship to patient		
Husband	33 (12.0)	30 (11.0)
Wife	63 (23.0)	69 (25.3)
Daughter	120 (43.8)	113 (41.4)
Other^b^	58 (21.2)	61 (22.3)
Morbidity		
Cardiovascular	101 (36.9)	122 (44.7)
Respiratory	54 (19.7)	40 (14.7)
Mental health	53 (19.3)	56 (20.5)
Musculoskeletal	135 (49.3)	142 (52.0)
Neurological	14 (5.1)	17 (6.2)
Cancer, all types	14 (5.1)	20 (7.3)
No. of prescribed medications		
0	90 (32.8)	68 (24.9)
1-2	93 (33.9)	110 (40.3)
3-4	55 (20.1)	54 (19.8)
>4	36 (13.2)	41 (15.0)
Educational level		
Primary	6 (2.2)	13 (4.8)
Secondary	93 (33.9)	115 (42.1)
Trade or diploma	60 (21.9)	60 (22.0)
Tertiary	115 (42.0)	85 (31.1)
Employment status^c^		
Full-time	62 (22.6)	55 (20.1)
Part-time	64 (23.4)	48 (17.6)
Retired	122 (44.5)	137 (50.2)
Unemployed	26 (9.5)	33 (12.1)
Provide physical care	126 (46.0)	108 (39.6)
Physical care frequency		
Daily	99 (79.2)	86 (79.6)
Every few days	14 (11.2)	11 (10.2)
Weekly	8 (6.4)	5 (4.6)
≥Every few weeks	4 (3.2)	6 (5.7)
Provide emotional care^d^	265 (96.7)	261 (95.6)
Emotional care frequency		
Daily	210 (79.2)	210 (80.5)
Every few days	29 (10.9)	36 (13.8)
Weekly	17 (6.4)	13 (5.0)
≥Every few weeks	9 (3.5)	2 (0.8)
Other care responsibilities^e^	92 (33.6)	95 (34.8)
Provide housework	260 (94.9)	257 (94.1)
Housework frequency		
Daily	186 (71.5)	172 (66.9)
Every few days	38 (14.6)	43 (16.7)
Weekly	24 (9.2)	38 (14.8)
≥Every few weeks	12 (4.7)	4 (1.6)
Provide social support	172 (62.8)	148 (54.2)
Social support frequency		
Daily	33 (19.2)	37 (25.0)
Every few days	50 (29.1)	29 (19.6)
Weekly	47 (27.3)	59 (39.9)
≥Every few weeks	42 (24.3)	23 (15.5)
**Patients **		
Age, mean (SD), y	83.16 (7.04)	83.45 (7.20)
Sex		
Male	125 (45.6)	126 (46.2)
Female	149 (54.4)	147 (53.8)
Hospital LOS, median (IQR), d	6.0 (4.0-10.0)	6.0 (4.0-11.0)
Place of residence^f^		
With caregiver^g^	172 (62.8)	175 (64.1)
Alone	71 (25.9)	75 (27.5)
With someone else	31 (11.3)	23 (8.4)

Abbreviation: LOS, length of stay.

^a^ Other birthplace included Croatia, Germany, Greece, Iran, Macedonia, Myanmar, Papua New Guinea, Singapore, US, or Zimbabwe.

^b^ Other relationship to patient included daughter-in-law, grandchild, partner, friend, sibling, niece, or nephew.

^c^ Employment status included self-employment.

^d^ Emotional care included providing comfort, reassurance, and validation and responding to feelings.

^e^ Other care responsibilities included providing physical, emotional, social, and domestic (housework) support.

^f^ At the time of hospital admission.

^g^ Person answering this question.

The intervention (FECH^+^ plus usual care) was delivered either partially or completely to 274 participants (100%) in the intervention group (eTable 1 in Supplement 2). The 6 planned telephone calls were made to 222 participants (81.0%) in the intervention group, with 261 (95.3%) receiving at least 4 calls. The median (IQR) time of all 6 calls was 122 (83-184) minutes, with a total median (IQR) time of the intervention (nurse preparation, gathering resources, and telephone call) being 272 (176-391) minutes.

### Primary Outcomes 

#### AQOL-8D Utility Score

Changes in caregivers’ HRQOL between the intervention and control group at baseline, 6 months (primary outcome), and 12 months (1 secondary outcome) are presented in Table 2 as well as eFigure 2 and eTable 5 in Supplement 2. At 6 months, the intervention group showed a small increase in HRQOL (measured with the AQOL-8D) compared with the control group, but the difference was not significant (between-group difference in AQOL-8D score, 0.01; 95% CI, −0.02 to 0.03; *P* = .62). The difference in AQOL-8D score between groups at 12 months was not statistically significant.

**Table 2.** Changes in Health-Related Quality of Life

Measure	Intervention group, mean (95% CI)	Change from baseline within group, mean (95% CI)	*P* value^a^	Control group, mean (95% CI)	Change from baseline within group, mean (95% CI)	*P* value^a^	Difference between groups, mean (95% CI)	*P* value for cross-sectional mean difference	*P* value^b^
**AQOL-8D utility score**^c^									
Baseline	0.75 (0.73 to 0.76)	NA	NA	0.74 (0.73 to 0.76)	NA	NA	0.01 (−0.01 to 0.03)	.59	NA
3 mo	0.76 (0.74 to 0.78)	0.01 (−0.01 to 0.03)	.23	0.74 (0.72 to 0.75)	−0.01 (−0.03 to 0.01)	.27	0.02 (−0.00 to 0.05)	.07	.10
6 mo	0.76 (0.74 to 0.77)	0.01 (−0.01 to 0.03)	.33	0.75 (0.73 to 0.77)	0.01 (−0.01 to 0.03)	.33	0.01 (−0.02 to 0.03)	.62	>.99
12 mo	0.75 (0.73 to 0.77)	0.00 (−0.02 to 0.02)	.96	0.74 (0.72 to 0.76)	−0.01 (−0.03 to 0.01)	.41	0.01 (−0.01 to 0.04)	.33	.54
**AQOL-8D psychosocial superdimension score^c^**									
Baseline	0.42 (0.40 to 0.44)	NA	NA	0.41 (0.39 to 0.43)	NA	NA	0.01 (−0.02 to 0.04)	.63	NA
3 mo	0.43 (0.41 to 0.45)	0.01 (−0.00 to 0.03)	.10	0.41 (0.38 to 0.43)	−0.01 (−0.02 to 0.01)	.42	0.03 (−0.00 to 0.06)	.07	.08
6 mo	0.44 (0.42 to 0.46)	0.02 (0.00 to 0.04)	.04	0.42 (0.40 to 0.44)	0.01 (−0.01 to 0.03)	.25	0.02 (−0.02 to 0.05)	.35	.55
12 mo	0.43 (0.41 to 0.46)	0.01 (−0.01 to 0.03)	.17	0.41 (0.39 to 0.43)	−0.00 (−0.02 to 0.02)	.84	0.02 (−0.01 to 0.06)	.18	.26

Abbreviations: AQOL-8D, Assessment of Quality of Life 8-Dimension; NA, not applicable.

^a^ Within-group change from baseline.

^b^ Interaction effect (difference of the differences between groups).

^c^ Score range: 0-1, with higher scores indicating better health-related quality of life.

#### AQOL-8D Psychosocial Superdimension

Changes in the HRQOL measured by the AQOL-8D psychosocial superdimension at baseline, 6 months, and 12 months are presented in Table 2 and eFigure 3 in Supplement 2. There were no significant differences between the 2 groups at 6 and 12 months. The intervention group demonstrated a significant within-group increase in the AQOL-8D psychosocial dimension at 6 months compared with baseline (mean score, 0.44 [95% CI, 0.42-0.46] vs 0.42 [95% CI, 0.40-0.44]; mean difference, 0.02 [95% CI, 0.00-0.04], *P* = .04). Changes in the psychosocial subdimensions (mental health, happiness, self-worth, coping, and relationships) are presented in eFigure 4, eFigure 5, and eTable 2 in Supplement 2. The intervention group showed a significant improvement in relationships at 12 months compared with the control group (mean score, 0.77 [95% CI, 0.75-0.80] vs 0.74 [95% CI, 0.74-0.76]; mean difference, −0.04; 95% CI, −0.07 to −0.01; *P* = .008]). There were significant within-group improvements in mental health in both the intervention and control groups at 6 months (mean difference, 0.02 [95% CI, 0.01-0.04; *P* = .001] and 0.02 [95% CI, 0.01-0.03; *P* = .001]) and 12 months (mean difference, 0.02 [95% CI, 0.00-0.03; *P* = .04] and 0.02 [95% CI, 0.00-0.03; *P* = .01]). There were significant within-group decreases in happiness at 12 months in both the intervention (mean difference, −0.03; 95% CI, −0.05 to −0.00; *P* = .03) and control (mean difference, −0.03; 95% CI, −0.05 to −0.00; *P* = .02) groups (due to rounding, values differ slightly from those shown in eTable 2 in Supplement 2).

#### AQOL-8D Physical Superdimension

There were no significant changes in the AQOL-8D physical superdimension or subdimensions (independent living, senses, and pain) between the 2 groups (eFigure 6, eTable 3, eFigure 7, and eTable 4 in Supplement 2). There was a within-group decrease in independent living at 12 months in both the intervention group (mean difference in scores, –0.02; 95% CI, –0.04 to –0.01; *P* = .01) and control group (mean difference in scores, –0.03; 95% CI, –0.04 to –0.01; *P* = .004) (due to rounding, values differ slightly from those shown in eTable 4 in Supplement 2).

### Secondary Outcomes

#### Preparedness to Care

**Figure 2.**
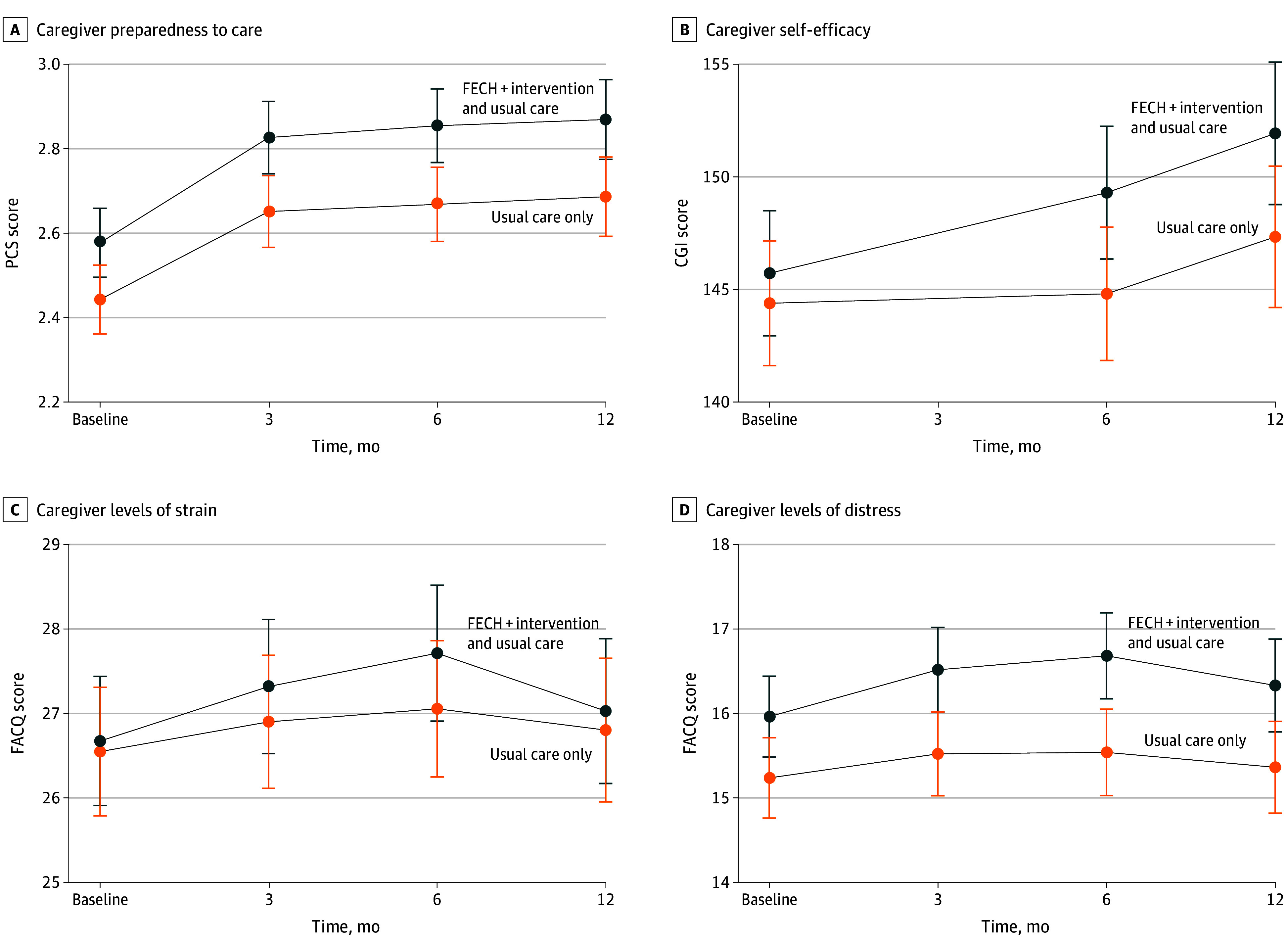
Secondary Outcomes Preparedness to care was measured with the Preparedness for Caregiving Scale (PCS; score range, 0-4, with higher scores indicating greater preparedness for caregiving). Self-efficacy was assessed with the Caregiver Inventory (CGI; score range, 21-189, with higher scores indicating better self-efficacy). Levels of strain and distress were measured with the Family Appraisal of Caregiving Questionnaire (FACQ; score range, 5-25 (strain) and 8 to 30 (distress), with higher scores indicating lower levels of strain and distress. Error bars represent 95% CIs.

Changes in caregivers’ preparedness to care (assessed using the PCS) at baseline, 6 months, and 12 months are presented in Figure 2, Table 3, and eTable 6 in Supplement 2. The intervention group was significantly more prepared to care at 6 months (mean score, 2.85 [95% CI, 2.77-2.94] vs 2.67 [95% CI, 2.58-2.76]; mean difference, 0.19 [95% CI, 0.06-0.31; *P* = .003]) and 12 months (mean score, 2.87 [95% CI, 2.77-2.96] vs 2.69 [95% CI, 2.59-2.78]; mean difference, 0.18 [95% CI, 0.05-0.32; *P* = .007]) after discharge compared with the control group. The difference of the differences between the groups at 6 and 12 months was not significant.

**Table 3.** Changes in Caregivers’ Preparedness to Care, Self-Efficacy, and Levels of Strain and Distress

Outcome	Mean score (95% CI)		*P* value^a^	Mean score (95% CI)		*P* value^a^	Difference between groups, mean score (95% CI)	*P* value for cross-sectional mean score difference	*P* value^b^
	Intervention group	Change from baseline score within group		Control group	Change from baseline score within group				
**Preparedness to care^c^**									
Baseline	2.58 (2.50 to 2.66)	NA	NA	2.44 (2.36 to 2.52)	NA	NA	0.13 (0.02 to 0.25)	.02	NA
3 mo	2.83 (2.74 to 2.91)	0.25 (0.17 to 0.33)	<.001	2.65 (2.57 to 2.74)	0.21 (0.13 to 0.29)	<.001	0.18 (0.05 to 0.30)	.005	.49
6 mo	2.85 (2.77 to 2.94)	0.28 (0.19 to 0.36)	<.001	2.67 (2.58 to 2.76)	0.23 (0.14 to 0.31)	<.001	0.19 (0.06 to 0.31)	.003	.39
12 mo	2.87 (2.77 to 2.96)	0.29 (0.20 to 0.38)	<.001	2.69 (2.59 to 2.78)	0.24 (0.15 to 0.33)	<.001	0.18 (0.05 to 0.32)	.007	.46
**Self-efficacy^d^**									
Baseline	145.72 (142.94 to 148.50)	NA	NA	144.39 (141.62 to 147.15)	NA	NA	1.34 (−2.60 to 5.27)	.51	NA
6 mo	149.30 (146.35 to 152.24)	3.58 (1.05 to 6.11)	.006	144.81 (141.85 to 147.77)	0.42 (−2.14 to 2.98)	.75	4.49 (0.29 to 8.69)	.04	.006
12 mo	151.93 (148.77 to 155.09)	6.21 (3.43 to 8.99)	<.001	147.34 (144.20 to 150.47)	2.95 (0.19 to 5.71)	.04	4.60 (0.12 to 9.07)	.04	<.001
**Levels of strain^e^**									
Baseline	26.67 (25.91 to 27.44)	NA	NA	26.55 (25.79 to 27.31)	NA	NA	0.13 (−0.96 to 1.21)	.82	NA
3 mo	27.32 (26.52 to 28.11)	0.65 (−0.01 to 1.30)	.054	26.90 (26.11 to 27.69)	0.35 (−0.30 to 1.00)	.29	0.42 (−0.71 to 1.54)	.47	.53
6 mo	27.71 (26.91 to 28.52)	1.04 (0.37 to 1.71)	.002	27.06 (26.25 to 27.86)	0.51 (−0.17 to 1.18)	.14	0.66 (−0.49 to 1.80)	.26	.27
12 mo	27.03 (26.17 to 27.89)	0.36 (−0.38 to 1.09)	.34	26.80 (25.95 to 27.65)	0.25 (−0.47 to 0.98)	.49	0.23 (−0.99 to 1.44)	.72	.85
**Levels of distress^e^**									
Baseline	15.96 (14.48 to 16.44)	NA	NA	15.24 (14.76 to 15.71)	NA	NA	0.73 (0.05 to 1.40)	.04	NA
3 mo	16.52 (16.02 to 17.02)	0.55 (0.10 to 1.01)	.02	15.52 (15.03 to 16.02)	0.29 (−0.17 to 0.74)	.22	0.99 (0.29 to 1.70)	.006	.42
6 mo	16.68 (16.17 to 17.19)	0.72 (0.25 to 1.19)	.003	15.54 (15.03 to 16.05)	0.30 (−0.17 to 0.78)	.21	1.14 (0.42 to 1.87)	.002	.22
12 mo	16.33 (15.78 to 16.88)	0.37 (−0.14 to 0.88)	.16	15.36 (14.82 to 15.91)	0.13 (−0.38 to 0.63)	.63	0.97 (0.19 to 1.74)	.01	.51

Abbreviation: NA, not applicable.

^a^ Within-group change from baseline.

^b^ Interaction effect (difference of the differences between groups).

^c^ Assessed using the Preparedness for Caregiving Scale: score range of 0 to 4, with higher scores indicating greater preparedness for caregiving.

^d^ Assessed using the Caregiver Inventory: score range of 21 to 189, with higher scores indicating better self-efficacy.

^e^ Assessed using the Family Appraisal of Caregiving Questionnaire: score range of 5 to 25 (strain) and 8 to 30 (distress), with higher scores indicating lower levels of strain and distress.

#### Self-Efficacy

Changes in caregivers’ self-efficacy (assessed using the CGI) at baseline, 6 months, and 12 months are presented in Figure 2 and Table 3. The intervention group showed significantly more self-efficacy to care at 6 months (mean score, 149.30 [95% CI, 146.35-152.24] vs 144.81 [95% CI, 141.85-147.77]) and 12 months (mean score, 151.93 [95% CI, 148.77-155.09] vs 147.34 [95% CI, 144.20-150.47]) after discharge compared with the control group. The difference of the differences between the groups at 6 months (mean difference, 4.49; 95% CI, 0.29-8.69; *P* = .04) and 12 months (mean difference, 4.60; 95% CI, 0.12 to 9.07; *P* = .04) were significant.

#### Levels of Strain and Distress

Changes over time in caregivers’ levels of strain and distress (assessed using the FACQ) at baseline, 6 months, and 12 months are presented in Figure 2 and Table 3. The intervention group was significantly less distressed at 6 months (mean score, 16.68 [95% CI, 16.17-17.19] vs 15.54 [95% CI, 15.03-16.05]) and 12 months (mean score, 16.33 [95% CI, 15.78-16.88] vs 15.36 [95% CI, 14.82-15.91]) after discharge compared with the control group. The difference of the differences between the groups at 6 and 12 months was not significant.

## Discussion

The FECH^+^ program did not significantly improve caregivers’ HRQOL. These results are consistent with systematic review findings that postdischarge support for caregivers of older adults at hospital discharge does not improve caregiver HRQOL.^29^ A Cochrane review^52^ concluded that telephone support as a sole intervention makes little or no difference to caregivers’ HRQOL,^52^ but a cluster randomized clinical trial that provided intensive face-to-face education and problem-solving for caregivers of adults with dementia^53^ also did not improve caregiver well-being, although it enhanced caregivers’ mood and reduced depressive symptoms.^53^ For caregivers of older adults, HRQOL is influenced by other factors, including the subjective burden of caregiving, resilience, patients’ dependence level, female sex, level of support, and available finances.^12,13,54,55^ More specific interventions tailored to individual populations of caregivers may be required. Reviews have found inconclusive evidence of the effectiveness of interventions to improve health and well-being of older caregivers and suggested identifying groups of caregivers who are most vulnerable to the adverse effect of caregiving and avoiding a one-size-fits-all approach to interventions.^31,56^ For example, specifically focusing on dyads of caregivers and older adults with dementia, a recent trial reported that delivering supportive calls over 12 months effectively increased attainment of personalized goals.^57^ However, caregivers of older patients have identified stressors that are specific to the time of a patient’s hospital discharge, including inadequate communication and low preparedness to care.^19^ This finding suggests that specific interventions that coordinate care, optimize communication, and provide sufficient services for caregivers at the time of discharge are required in addition to preadmission support.^19^

Additionally, interventions that include psychological, financial, social, and home care support may improve caregivers’ HRQOL when patients are discharged. A scoping review found that holistic support is needed to maintain caregivers’ physical and emotional health for their roles when older adults are discharged from the hospital.^58^ National caregiver organizations have emphasized the need for practical, financial, and emotional support in sustaining the caregiving role.^1,2^ An analysis of a national database identified that financial subsidies and home care support increased caregivers’ life satisfaction by 10% to 15%.^59^

The FECH^+^ program significantly increased caregivers’ preparedness to care and self-efficacy and reduced distress from caring at 6 and 12 months after hospital discharge. Differences in preparedness to care between groups were only slightly less than the 0.25 points that we determined to be clinically meaningful in a pilot trial.^32^ However, that difference was observed at 3 weeks after discharge. In the present trial, the differences between the groups of 0.18 to 0.19 points were found at the longer time points of 6 and 12 months. Improving preparedness to care is associated with lower levels of depression, strain, and burden and higher levels of resilience and good mood.^55,60,61^ The intervention group showed increased caregiver self-efficacy and reduced levels of distress compared with the control group. Caregiver self-efficacy is inversely related to caregiver strain and depression,^45,62,63,64^ which suggests that caregivers who develop better coping and problem-solving skills are more likely to report a decrease in objective burden and stress or anxiety.^65,66,67^ These findings are consistent with those of a 12-month trial of a supportive intervention for caregivers of older adults with dementia that showed improved caregiver self-efficacy and reduced burden and depression at 6 months.^68^

### Limitations

A study limitation was that due to COVID-19 pandemic restrictions, recruitment ceased early in the Queensland sites, restricting generalizability. Another limitation was that we did not provide an intervention to patients along with the caregiver intervention,^68^ which might have altered the results. We did not evaluate caregiver health literacy, and poor caregiver health literacy is associated with difficulty in understanding instructions, communicating with health professionals, and managing health care problems.^69^ Testing of multiple outcomes in 1 trial runs a slight risk of erroneous rejection of a null hypothesis; however, we noted that the direction of effect was consistent across all secondary outcomes, with the CIs showing plausible ranges of effects.

## Conclusions

In this randomized clinical trial, nurse telephone support for caregivers of older patients after hospital discharge did not significantly improve caregivers’ HRQOL in the 12 months after discharge compared with usual care alone. Further research is needed into how to improve caregivers’ HRQOL when their older care recipient is discharged from the hospital.

## Notes

Supplement 1.
Trial Protocol

Supplement 2. **eTable 1.** TIDieR Checklist for FECH^+^ Intervention **eFigure 1.** Structure of the AQoL-8D **eMethods.** Secondary Outcome Measures **eFigure 2.** Changes in Health-Related Quality of Life AQoL-8D: Overall Utility Score **eFigure 3.** Changes in Health-Related Quality of Life AQoL-8D: Psychosocial Super Dimension **eFigure 4.** Changes in Health-Related Quality of Life AQoL-8D: Psychosocial Subscales a) Mental Health; b) Happiness; c) Self-Worth **eFigure 5.** Changes in Health-Related Quality of Life AQoL-8D: Psychosocial Subscales d) Coping; e) Relationships **eTable 2.** Changes in Health-Related Quality of Life AQoL-8D: Psychosocial Subscales a) Mental Health; b) Happiness; c) Self-Worth; d) Coping; e) Relationships **eFigure 6.** Changes in Health-Related Quality of Life AQoL-8D: Physical Super Dimension **eTable 3.** Changes in Health-Related Quality of Life AQoL-8D: Physical Super Dimension **eFigure 7.** Changes in Health-Related Quality of Life AQoL-8D: Physical Subscales a) Independent Living; b) Senses; c) Pain **eTable 4.** Changes in Health-Related Quality of Life AQoL-8D: Physical Subscales a) Independent Living; b) Senses; c) Pain **eTable 5.** AQoL-8D: Summary Statistics **eTable 6.** Secondary Outcomes Summary Statistics: Preparedness for Care (PCS), Caregiver Inventory (CGI), Family Appraisal of Caregiving Questionnaire (FACQ)

Supplement 3.
Data Sharing Statement

## References

[1] 2020 Report: caregiving in the US. National Alliance for Caregiving; AARP. Accessed March 30, 2024. https://www.caregiving.org/wp-content/uploads/2021/01/full-report-caregiving-in-the-united-states-01-21.pdf

[2] Petrillo M, Bennet M. Valuing carers 2021: England and Wales. Accessed March 30, 2024. https://www.carersuk.org/reports/valuing-carers-research-report/

[3] Schirmer J, Mylek M, Miranti R. Caring for Others and Yourself. Canberra, ACT: Carer’s Australia and University of Canberra; 2022. Accessed March 30, 2024. https://www.carersaustralia.com.au/wp-content/uploads/2021/10/211011_Carer-Wellbeing-Survey_Final.pdf

[4] Reinhard SC, Caldera S, Houser A, Choula RB. Valuing the invaluable: 2023 update: strengthening supports for family caregivers. Accessed March 30, 2024. https://www.aarp.org/content/dam/aarp/ppi/2023/3/valuing-the-invaluable-2023-update.doi.10.26419-2Fppi.00082.006.pdf

[5] Unpaid carers: a silent workforce. Lancet Public Health. 2024;9(1):e1. doi:10.1016/S2468-2667(23)00311-0 38176836

[6] Leykum LK, Penney LS, Dang S, . Recommendations to improve health outcomes through recognizing and supporting caregivers. J Gen Intern Med. 2022;37(5):1265-1269. doi:10.1007/s11606-021-07247-w 34981348 PMC8722428

[7] Mather M, Scommegna P. Fact sheet: aging in the United States. Accessed March 30, 2024. https://www.prb.org/resources/fact-sheet-aging-in-the-united-states/#:~:text=The%20number%20of%20Americans%20ages,from%2017%25%20to%2023%25

[8] Older Australians. Australian Institute of Health and Welfare. Accessed March 30, 2024. https://www.aihw.gov.au/reports/older-people/older-australians/contents/about

[9] Ageing and health. World Health Organization. Accessed March 30, 2024. https://www.who.int/news-room/fact-sheets/detail/ageing-and-health

[10] Pristavec T. The burden and benefits of caregiving: a latent class analysis. Gerontologist. 2019;59(6):1078-1091. doi:10.1093/geront/gny022 29659788 PMC6858826

[11] Kotwal AA, Allison TA, Halim M, . “Relationships, very quickly, turn to nothing”: loneliness, social isolation, and adaptation to changing social lives among persons living with dementia and care partners. Gerontologist. 2024;64(4):gnae014. doi:10.1093/geront/gnae014 38499400 PMC10948338

[12] Furnival A, Cullen D. Caring costs us: the economic impact on lifetime income and retirement savings of informal carers—a report for carers Australia. Accessed March 30, 2024. https://www.carersaustralia.com.au/wp-content/uploads/2022/04/Final-Economic-impact-income-and-retirement-Evaluate-Report-March-2022.pdf

[13] Bom J, Bakx P, Schut F, van Doorslaer E. The impact of informal caregiving for older adults on the health of various types of caregivers: a systematic review. Gerontologist. 2019;59(5):e629-e642. doi:10.1093/geront/gny137 30395200 PMC6850889

[14] Di Lorito C, Bosco A, Godfrey M, . Mixed-methods study on caregiver strain, quality of life, and perceived health. J Alzheimers Dis. 2021;80(2):799-811. doi:10.3233/JAD-201257 33579842 PMC8075381

[15] Pozet A, Darnis S, Bonnet M, . Quality of life and needs in caregivers: results from the prospective multicentric open-label randomized study of informal caregivers of elderly patients. Int J Public Health. 2023;68:1605459. doi:10.3389/ijph.2023.1605459 37711159 PMC10498993

[16] Jeste DV, Mausbach B, Lee EE. Caring for caregivers/care partners of persons with dementia. Int Psychogeriatr. 2021;33(4):307-310. doi:10.1017/S1041610221000557 33970060 PMC8752059

[17] Penning MJ, Wu Z. Caregiver stress and mental health: impact of caregiving relationship and gender. Gerontologist. 2016;56(6):1102-1113. doi:10.1093/geront/gnv038 26035875

[18] Lilleheie I, Debesay J, Bye A, Bergland A. Informal caregivers’ views on the quality of healthcare services provided to older patients aged 80 or more in the hospital and 30 days after discharge. BMC Geriatr. 2020;20(1):97. doi:10.1186/s12877-020-1488-1 32164569 PMC7068939

[19] Liebzeit D, Jaboob S, Bjornson S, . A scoping review of unpaid caregivers’ experiences during older adults’ hospital-to-home transitions. Geriatr Nurs. 2023;53:218-226. doi:10.1016/j.gerinurse.2023.08.010 37598425

[20] Callister C, Jones J, Schroeder S, . Caregiver experiences of care coordination for recently discharged patients: a qualitative metasynthesis. West J Nurs Res. 2020;42(8):649-659. doi:10.1177/0193945919880183 31585516 PMC7124970

[21] Hill AM, McPhail SM, Haines TP, . Falls after hospital discharge: a randomized clinical trial of individualized multimodal falls prevention education. J Gerontol A Biol Sci Med Sci. 2019;74(9):1511-1517. doi:10.1093/gerona/glz026 30721940 PMC7330456

[22] Auerbach AD, Kripalani S, Vasilevskis EE, . Preventability and causes of readmissions in a national cohort of general medicine patients. JAMA Intern Med. 2016;176(4):484-493. doi:10.1001/jamainternmed.2015.7863 26954564 PMC6900926

[23] Andrew MK, MacDonald S, Godin J, . Persistent functional decline following hospitalization with influenza or acute respiratory illness. J Am Geriatr Soc. 2021;69(3):696-703. doi:10.1111/jgs.16950 33294986 PMC7984066

[24] Hunt-O’Connor C, Moore Z, Patton D, Nugent L, Avsar P, O’Connor T. The effect of discharge planning on length of stay and readmission rates of older adults in acute hospitals: a systematic review and meta-analysis of systematic reviews. J Nurs Manag. 2021;29(8):2697-2706. doi:10.1111/jonm.13409 34216502

[25] Gonçalves-Bradley DC, Lannin NA, Clemson L, Cameron ID, Shepperd S. Discharge planning from hospital. Cochrane Database Syst Rev. 2022;2(2):CD000313. doi:10.1002/14651858 35199849 PMC8867723

[26] Mabire C, Dwyer A, Garnier A, Pellet J. Meta-analysis of the effectiveness of nursing discharge planning interventions for older inpatients discharged home. J Adv Nurs. 2018;74(4):788-799. doi:10.1111/jan.13475 28986920

[27] van den Broek S, Westert GP, Hesselink G, Schoon Y. Effect of ED-based transitional care interventions by healthcare professionals providing transitional care in the emergency department on clinical, process and service use outcomes: a systematic review. BMJ Open. 2023;13(3):e066030. doi:10.1136/bmjopen-2022-066030 36918249 PMC10016244

[28] Toles M, Preisser JS, Colón-Emeric C, . Connect-Home transitional care from skilled nursing facilities to home: a stepped wedge, cluster randomized trial. J Am Geriatr Soc. 2023;71(4):1068-1080. doi:10.1111/jgs.18218 36625769 PMC10089938

[29] Smith TO, Pearson M, Pfeiffer K, Crotty M, Lamb SE. Caregiver interventions for adults discharged from the hospital: systematic review and meta-analysis. J Am Geriatr Soc. 2019;67(9):1960-1969. doi:10.1111/jgs.16048 31350918

[30] Pritchard E, Cussen A, Delafosse V, Swift M, Jolliffe L, Yeates H. Interventions supporting caregiver readiness when caring for patients with dementia following discharge home: a mixed-methods systematic review. Australas J Ageing. 2020;39(3):e239-e250. doi:10.1111/ajag.12765 31944506

[31] Spiers GF, Liddle J, Kunonga TP, . What are the consequences of caring for older people and what interventions are effective for supporting unpaid carers? a rapid review of systematic reviews. BMJ Open. 2021;11(9):e046187. doi:10.1136/bmjopen-2020-046187 34588234 PMC8483048

[32] Toye C, Parsons R, Slatyer S, . Outcomes for family carers of a nurse-delivered hospital discharge intervention for older people (the Further Enabling Care at Home Program): single blind randomised controlled trial. Int J Nurs Stud. 2016;64:32-41. doi:10.1016/j.ijnurstu.2016.09.012 27684320

[33] Hill AM, Moorin R, Slatyer S, . Evaluating the provision of Further Enabling Care at Home (FECH+) for informal caregivers of older adults discharged home from hospital: protocol for a multicentre randomised controlled trial. BMJ Open. 2021;11(6):e046600. doi:10.1136/bmjopen-2020-046600 34155075 PMC8217916

[34] Moher D, Hopewell S, Schulz KF, ; Consolidated Standards of Reporting Trials Group. CONSORT 2010 explanation and elaboration: updated guidelines for reporting parallel group randomised trials. J Clin Epidemiol. 2010;63(8):e1-e37. doi:10.1016/j.jclinepi.2010.03.004 20346624

[35] Who is a carer? Carers Australia. Accessed March 30, 2024. https://www.carersaustralia.com.au/about-carers/who-is-a-carer/

[36] Hoffmann TC, Glasziou PP, Boutron I, . Better reporting of interventions: Template for Intervention Description and Replication (TIDieR) checklist and guide. BMJ. 2014;348:g1687. doi:10.1136/bmj.g1687 24609605

[37] Beaudreau SA, Gould CE, Sakai E, Huh JWT. Problem-solving therapy. In: Pachana N, ed. Encyclopedia of Geropsychology. Springer; 2015. doi:10.1007/978-981-287-080-3_90-1

[38] Nezu AM, Nezu CM, D’Zurilla TJ. Solving Life’s Problems: a 5-Step Guide to Enhanced Well-Being. Springer Publishing Co; 2007.

[39] Ewing G, Grande G; National Association for Hospice at Home. Development of a Carer Support Needs Assessment Tool (CSNAT) for end-of-life care practice at home: a qualitative study. Palliat Med. 2013;27(3):244-256. doi:10.1177/0269216312440607 22450160

[40] Patchwood E, Woodward-Nutt K, Rhodes SA, . Organising Support for Carers of Stroke Survivors (OSCARSS): a cluster randomised controlled trial with economic evaluation. BMJ Open. 2021;11(1):e038777. doi:10.1136/bmjopen-2020-038777 33436463 PMC7805348

[41] Richardson J, Iezzi A, Khan MA, Maxwell A. Validity and reliability of the Assessment of Quality of Life (AQoL)-8D multi-attribute utility instrument. Patient. 2014;7(1):85-96. doi:10.1007/s40271-013-0036-x 24271592 PMC3929769

[42] Richardson J, Khan MA, Iezzi A, Maxwell A. Comparing and explaining differences in the magnitude, content, and sensitivity of utilities predicted by the EQ-5D, SF-6D, HUI 3, 15D, QWB, and AQoL-8D multiattribute utility instruments. Med Decis Making. 2015;35(3):276-291. doi:10.1177/0272989X14543107 25159172

[43] Archbold PG, Stewart BJ, Greenlick MR, Harvath T. Mutuality and preparedness as predictors of caregiver role strain. Res Nurs Health. 1990;13(6):375-384. doi:10.1002/nur.4770130605 2270302

[44] Zwicker D. Preparedness for Caregiving Scale. The Hartford Institute for Geriatric Nursing, New York University Rory Meyers College of Nursing. 2018. Accessed June 30, 2024. https://hign.org/consultgeri/try-this-series/preparedness-caregiving-scale

[45] Serpentini S, Guandalini B, Tosin G, . Assessment of self-efficacy for caregiving in oncology: Italian validation of the caregiver inventory (CGI-I). BMC Palliat Care. 2021;20(1):166. doi:10.1186/s12904-021-00849-5 34670541 PMC8529803

[46] Merluzzi TV, Philip EJ, Vachon DO, Heitzmann CA. Assessment of self-efficacy for caregiving: the critical role of self-care in caregiver stress and burden. Palliat Support Care. 2011;9(1):15-24. doi:10.1017/S1478951510000507 21352614

[47] Cooper B, Kinsella GJ, Picton C. Development and initial validation of a family appraisal of caregiving questionnaire for palliative care. Psychooncology. 2006;15(7):613-622. doi:10.1002/pon.1001 16287207

[48] Mahoney FI, Barthel DW. Functional evaluation: the Barthel index. Md State Med J. 1965;14:61-65. 14258950

[49] Aoun SM, Monterosso L, Kristjanson LJ, McConigley R. Measuring symptom distress in palliative care: psychometric properties of the Symptom Assessment Scale (SAS). J Palliat Med. 2011;14(3):315-321. doi:10.1089/jpm.2010.0412 21254812

[50] Faul F, Erdfelder E, Buchner A, Lang AG. Statistical power analyses using G*Power 3.1: tests for correlation and regression analyses. Behav Res Methods. 2009;41(4):1149-1160. doi:10.3758/BRM.41.4.1149 19897823

[51] Tukey JW. Exploratory Data Analysis. Addison-Wesley Pub. Co; 1997.

[52] Corry M, Neenan K, Brabyn S, Sheaf G, Smith V. Telephone interventions, delivered by healthcare professionals, for providing education and psychosocial support for informal caregivers of adults with diagnosed illnesses. Cochrane Database Syst Rev. 2019;5(5):CD012533. doi:10.1002/14651858.CD012533.pub2 31087641 PMC6516056

[53] Gitlin LN, Roth DL, Marx K, . Embedding caregiver support within adult day services: outcomes of a multisite trial. Gerontologist. 2024;64(4):gnad107. doi:10.1093/geront/gnad107 37549428 PMC10943495

[54] Vrettos I, Anagnostopoulos F, Voukelatou P, . Factors associated with health-related quality of life of informal caregivers of older patients and the mediating role of subjective caregivers’ burden. Psychogeriatrics. 2023;23(2):286-297. doi:10.1111/psyg.12930 36597270

[55] Gutierrez-Baena B, Romero-Grimaldi C. Predictive model for the preparedness level of the family caregiver. Int J Nurs Pract. 2022;28(3):e13057. doi:10.1111/ijn.13057 35388583 PMC9285821

[56] Kirvalidze M, Abbadi A, Dahlberg L, Sacco LB, Morin L, Calderón-Larrañaga A. Effectiveness of interventions designed to mitigate the negative health outcomes of informal caregiving to older adults: an umbrella review of systematic reviews and meta-analyses. BMJ Open. 2023;13(4):e068646. doi:10.1136/bmjopen-2022-068646 37085312 PMC10124259

[57] Cooper C, Vickerstaff V, Barber J, . A psychosocial goal-setting and manualised support intervention for independence in dementia (NIDUS-Family) versus goal setting and routine care: a single-masked, phase 3, superiority, randomised controlled trial. Lancet Healthy Longev. 2024;5(2):e141-e151. doi:10.1016/S2666-7568(23)00262-3 38310894 PMC10834374

[58] Allen J, Woolford M, Livingston PM, Lobchuk M, Muldowney A, Hutchinson AM. Informal carer support needs, facilitators and barriers in transitional care for older adults from hospital to home: a scoping review. J Clin Nurs. 2023;32(19-20):6773-6795. doi:10.1111/jocn.16767 37272211

[59] Costa-Font J, Vilaplana-Prieto C. Mental health effects of caregivers respite: subsidies or supports? J Econ Ageing. 2022;23(c):100398. doi:10.1016/j.jeoa.2022.100398

[60] Kuzmik A, Boltz M, Resnick B, BeLue R. Evaluation of the Caregiver Preparedness Scale in African American and White caregivers of persons with dementia during post-hospitalization transition. J Nurs Meas. 2021;JNM-D-20-00087. doi:10.1891/JNM-D-20-00087 34518397 PMC9514879

[61] Petruzzo A, Biagioli V, Durante A, . Influence of preparedness on anxiety, depression, and quality of life in caregivers of heart failure patients: testing a model of path analysis. Patient Educ Couns. 2019;102(5):1021-1028. doi:10.1016/j.pec.2018.12.027 30611564

[62] Giovannetti ER, Wolff JL, Xue QL, . Difficulty assisting with health care tasks among caregivers of multimorbid older adults. J Gen Intern Med. 2012;27(1):37-44. doi:10.1007/s11606-011-1831-5 21874385 PMC3250537

[63] Amer Nordin A, Mohd Hairi F, Choo WY, Hairi NN. Care recipient multimorbidity and health impacts on informal caregivers: a systematic review. Gerontologist. 2019;59(5):e611-e628. doi:10.1093/geront/gny072 29982539

[64] Pereira-Osorio C, Brickell E, Lee B, Arredondo B, Sawyer RJ. Performance of the modified caregiver strain index in a sample of Black and White persons living with dementia and their caregivers. Gerontologist. 2024;64(7):gnae052. doi:10.1093/geront/gnae052 38769644 PMC11181709

[65] Leung DYP, Chan HYL, Chiu PKC, Lo RSK, Lee LLY. Source of social support and caregiving self-efficacy on caregiver burden and patient’s quality of life: a path analysis on patients with palliative care needs and their caregivers. Int J Environ Res Public Health. 2020;17(15):5457. doi:10.3390/ijerph17155457 32751147 PMC7432213

[66] Duggleby W, Williams A, Ghosh S, . Factors influencing changes in health related quality of life of caregivers of persons with multiple chronic conditions. Health Qual Life Outcomes. 2016;14:81. doi:10.1186/s12955-016-0486-7 27229926 PMC4882862

[67] Garand L, Morse JQ, ChiaRebecca L, . Problem-solving therapy reduces subjective burden levels in caregivers of family members with mild cognitive impairment or early-stage dementia: secondary analysis of a randomized clinical trial. Int J Geriatr Psychiatry. 2019;34(7):957-965. doi:10.1002/gps.5095 30868641 PMC6579659

[68] Possin KL, Merrilees JJ, Dulaney S, . Effect of collaborative dementia care via telephone and internet on quality of life, caregiver well-being, and health care use: the care ecosystem randomized clinical trial. JAMA Intern Med. 2019;179(12):1658-1667. doi:10.1001/jamainternmed.2019.4101 31566651 PMC6777227

[69] O’Conor R, Bonham M, Magnuson G, . Caregiver health literacy and health task performance: findings from the LitCog caregiver cohort study. PEC Innov. 2023;3:100240. doi:10.1016/j.pecinn.2023.100240 38161686 PMC10757034

