# Supplementary material for: Nurse Telephone Support for Caregivers of Older Adults at Hospital Discharge: A Randomized Clinical Trial
Source: JAMA Netw Open. 2024 Oct 25;7(10):e2441019. doi: 10.1001/jamanetworkopen.2024.41019 (PMC11581515; doi:10.1001/jamanetworkopen.2024.41019)
Supplement: Supplement 1. — Trial Protocol [file jamanetwopen-e2441019-s001.pdf]

Multicentre randomised controlled trial: caregiver, patient, and system outcomes from a program supporting informal caregivers of older people discharged home from hospital

Evaluating the Further Enabling Care at Home (FECH) post-discharge program as a way to support carers of older hospital patients. Version 6: May 14 2020. **Summary**

Supporting home caregiving for older people in failing health is now a critical issue in Australia. Hospital admission of an older person receiving care from family or friends presents an opportunity to identify and address the support needs of these carers, and to build their skills to continue to do this independently. This study will trial the Further Enabling Care at Home (FECH) program, which was previously found to benefit carers of older people returning home from hospital in terms of reduced carer strain and distress and increased preparedness to care. The program is delivered by specially prepared nurses over the phone, after the discharge, and is designed to guide the carer to identify and rate their support needs, then address the most pressing of these needs via a problem solving approach. The intent is to build the capacity of carers over the 6-month program so that they can then identify and address their support needs without FECH nurse help.

Because caregiving preparedness was previously improved by the program, it is arguable that improvements in care, and thus in care recipients' independence and symptoms, may result, with subsequent reductions in care recipients' needs for health services and residential aged care. Similarly, improved health related quality of life (HRQoL) may occur in carers through completion of the FECH program, thus reducing their use of health services. These reductions in service use, and therefore in costs, could then help to justify widespread program implementation.

Aims of this study are to (a) measure impacts from the FECH program on carers (HRQoL, self-efficacy, preparedness, strain, distress), patients (symptom distress, independence), and the use and costs of health and residential care services and (b) explore and describe how caregiving is influenced by the program. The randomised controlled trial will be conducted in Queensland (Qld) and Western Australia (WA), involving three hospitals. Dyads (n=925) will be recruited at these hospitals. Each dyad will comprise an older person (aged 70+) being discharged home and their English speaking carer. Some patients will be included via a waiver of consent (Western Australia) or proxy consent (Queensland) because they lack the capacity to consider consenting. Each dyad will be randomly assigned to receive usual carer support (control group) or FECH program (for the carer) plus usual carer support (intervention group).

Data will be collected from all carers, by phone or via a web-based questionnaire (their choice), straight after the care recipient's discharge and then after 3, 6, and 12 months. Care recipients (discharged patients) will also be asked to rate their symptoms over the phone at these time points, with carer ratings accepted if care recipients are unable to do this. Carers will rate care recipients' independence. A proportion of carers will be included in telephone interviews about their experiences of, and responses to, the program, in particular, how it influences caregiving, until no new information is forthcoming. Information routinely collected about health service use will be included for carers and care recipients, accessed via

state and federal agencies. Carers will also report health service use by the older person receiving care and themselves, plus care recipient entry into a residential aged care facility (for respite or long-term care). Analyses will establish differences in outcomes between the FECH and control group, and cost outcomes for health and residential care.

Contents

Project Team Roles and Responsibilities 3
- Chief Investigators 3
- Associate Investigators 3
- Project Staff 4
- Collaborators and Other Contributors 4

Research Sites 4

Resources 4

Applications 4

Background 5
- Literature review 5
- Rationale for the Study 6

Aims and Hypothesis 8

Expected Outcomes 9

Project Design 10
- Methodological Approach 10
- Settings 11
- Control Condition (usual care) 12
- Intervention (FECH program + usual care) 12
- Participants 14
- Power Calculations 15
- Recruitment 16
- Database Setup, Random Assignment, and Blinding 21
- Outcome Measures and Demographic and Caregiving Details 22
- Data Collection or Gathering 23
- Research Activities and Timeline 25
- Impact of and Response to Participant Withdrawal 25
- Process Evaluation 26
- Data Linkage 27
- Ethical Issues 27
- Data Management 29

Statistical Analysis 35
Qualitative Analysis 36
Results, Outcomes and Future Plans 36
References 37

Project Team Roles and Responsibilities

Chief Investigators

A Professor Anne-Marie Hill The University of Western Australia, overseeing the whole project.

B Professor Wendy Moyle Griffith University:

Oversight of the Queensland project arm

C Associate Professor Rachael Moorin Curtin University:

Health economist role – oversight of accessing, managing, and analysing administrative health data

D Professor Keith Hill Monash University

Supporting oversight of quantitative study components

E Associate Professor Susan Slatyer Murdoch University:

Supporting oversight of qualitative study components

F Associate Professor Christina Bryant:

University of Melbourne. Oversight of intervention component

G Doctor Nicholas Waldron Armadale Health Service, WA:

Supporting oversight of quantitative study components

H Professor Samar Aoun La Trobe University:

Supporting oversight of carer related study components

Associate Investigators

Dr Ami Kamdar Sir Charles Gairdner Hospital:

Supporting study recruitment in that hospital setting

Ms Caroline Reberger Sir Charles Gairdner Hospital:

Supporting study recruitment in that hospital setting and providing FECH program input

Associate Professor Cindy Jones Bond University:

Supporting study conduct in Queensland

Associate Professor Laurie Grealish Griffith University:

Supporting study conduct in Queensland

Ms Mary Bronson Sir Charles Gairdner Hospital:

Supporting study recruitment in the hospital setting

Professor Max Bulsara Notre Dame University:

Supporting statistical analysis

Dr Sean Maher Sir Charles Gairdner Hospital:

Supporting study recruitment in the hospital setting

Project Staff

Ms Patricia (Trish) Starling Project Manager (WA) from January 2020):

Oversight of study in WA and liaison/communication with the Qld team.

The remaining staff members will be reconfirmed before the study restarts in 2021.

Collaborators and Other Contributors

Dr Helen Atkinson Executive Officer, Clinical Trials & Data Management Centre (CTDMC) at Curtin University: liaising with the project team regarded the agreed CTDMC arrangements for this study

Mr Howard Lance, Consumer Advocate, Consumer and Community Health Research Network, WA

Research Sites

In Queensland: Gold Coast Health Care Group (Robina Hospital and Gold Coast University Hospital), Griffith University. In Western Australia: Sir Charles Gairdner Hospital, Curtin University

Resources

Funding provided by NHMRC $1,500,381.

Applications

Applications are being made to the Sir Charles Gairdner Osborne Park Health Care Group Human Research Ethics Committee (Primary HREC), the HREC of the Department of Health of Western Australia (Specialist HREC), the HREC of the Gold Coast Health Care Group (National Mutual Acceptance), and the HRECs of Curtin University and Griffith University (recognition of prior approval). Administrative data are being sought from the Data Linkage Branch of WA Health, data linkage services of Queensland Health, and the Department of Human Services (DHS) (now Services Australia). Research governance approval is also required from Sir Charles Gairdner Hospital, Gold Coast Health, Curtin University, and Griffith University

Background

Literature review

Australia's improved life expectancy means that people turning 65 in 2015 were likely to live for another 20 years (1). However, there is a corresponding increase in the prevalence of chronic health conditions and disability in later life (2), with 10 of those extra years lived with disability (1) and 60% of people aged $\geq$ 65 having at least two chronic health conditions (3). With older Australians seeking to live and die at home (4, 5), the caregiving context may include disease progression, falls, frailty, symptom exacerbation, and acute-on-chronic illness preceding eventual death.

Caregiving risks. While there are a range of personal and societal benefits from caregiving, caregiving can jeopardise health. In a New South Wales (NSW) survey, almost 50% of informal carers provided more than 70 hours per week of care, with 33% providing care '24/7' (6). Yet the mean age of primary carers for older people in Australia is 55, and over 37% of these carers live with disability themselves (7); also, over 92% of carers responding to a national seniors' survey reported chronic illness (8); and, in NSW, 44% reported deteriorating health (6).

Mental health risks can be particularly severe. In a large survey in the Australian Capital Territory (ACT) (n=2,081), caregiving was associated with a greater than 50% increased risk of clinically significant anxiety and depression (9). In other studies, caregiving outcomes have included burden, stress, and role strain (10), with greater carer strain associated with lower Health Related Quality of Life (HRQoL) (11).

These risks can be ameliorated. Resources exist to help carers, and carer burden, stress, and depression, in particular, can be reduced via individualised supportive carer interventions (10). However, ensuring that resources and services are used by those who need them is problematic. The carer 'label' may be rejected because caregiving is viewed as part of the pre-existing relationship (12) or the carer seeks to 'hide' dependency in the care recipient (13). Access to support can also be challenging (14), evidenced by under 50% of NSW carers surveyed obtaining services (6).

Outcomes when carer health fails. Negative impacts from caregiving on HRQoL can lead the carer to relinquish care (15) and system costs from this are extremely high. A relatively small and temporary increase in needs can also precipitate otherwise avoidable hospitalisation (16). The mean cost of an Australian public hospital 'separation' in 2013-14 was $5,100 (17), and older people have longer hospital stays, often complicated by adverse events, with approximately 25% of additional costs relating to hospital acquired conditions (18). Unsurprisingly, after accounting for care recipient health status, carer distress predicts

Residential Aged Care Facility (RACF admission) (19). Australian governments spent over $11 billion on RACF admissions in 2015-6, with spending escalating by 19% over 5 years (20).

Hospitalisation as an opportunity to identify and support carers. Development of the FECH program evolved from our team's investigation of early, unplanned returns to hospital by older people discharged home from a Western Australian (WA) Medical Assessment Unit (MAU). By 7 days post-discharge 8.2% of patients had re-presented to an Emergency Department (ED) and more than 20% died within the 2-year follow-up period (21). When older patients who re-presented within 28 days (n=12), their family carers (n=15), and staff members (n=35) were interviewed: patients reported symptom distress, carers told of supporting patients in declining health, and the staff determined that carers needed more information on disease processes to mitigate symptom exacerbation (16). Hospital discharge plans can address issues such as symptom management but these are plans focussing on the patient (22). The FECH program offers a complementary, problem-solving, carer-focussed approach to improve outcomes for the carer and the patient.

Problem-solving is a practical step-by-step approach typically involving identifying and defining the problem, understanding it, setting goals and generating solutions, implementing a course of action, and evaluating its efficacy (23). The approach focuses on problems, not psychological symptoms, so it can be taught to, and implemented by, non-psychologists. Problem solving has been successfully used in other interventions to support carers of older people, including those delivered via telehealth (24).

Rationale for the Study

Figure 1 summarises potential benefits for carers, care recipients, and health and residential care systems from FECH program implementation. Relationships illustrated are derived from preliminary findings (25) plus other findings showing that:

(i) negative health impacts from caregiving are modulated when carer strain is reduced (11);

(ii) improved caregiving self-efficacy may accompany greater caregiving preparedness (26),

(iii) improved caregiving self-efficacy is also associated with better carer HRQoL (27), and

(iv) reduced risk of RACF admission is associated with lower levels of carer distress (19) (shown as occurring via improved HRQoL).

Assumptions, partly supported by findings (25), are that (i) better prepared carers will more effectively manage care recipients' symptoms and functional limitations and (ii) improved carer HRQoL and more effective care will translate into reduced health and residential care service use plus related reductions in system costs.

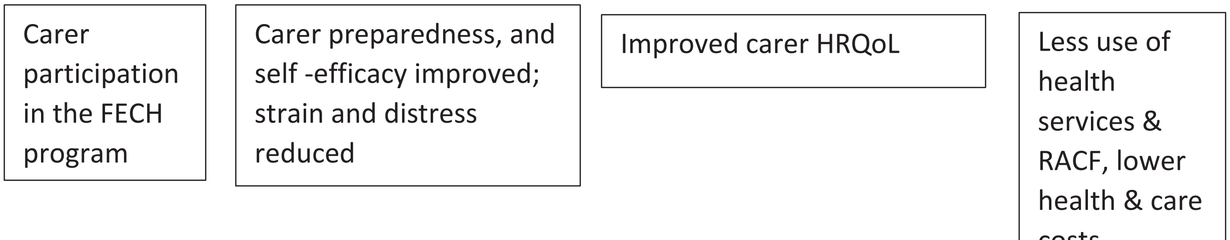

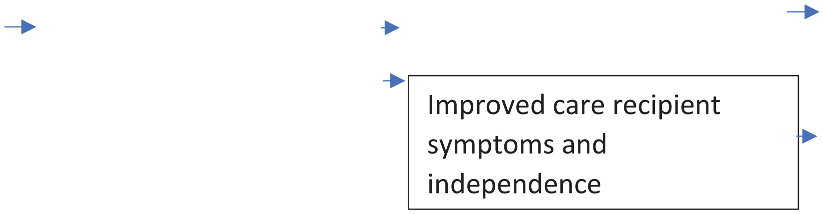

Figure 1. Conceptual framework

A single blind Randomised Controlled Trial (RCT) conducted by members of our team tested a brief (3-week) version of the FECH program after discharge from the MAU. This study documented the feasibility and cost of the program for carers (mean $AUD 268.28 per dyad (28)), and determined variation in effects for primary and secondary outcomes to inform plans for a more comprehensive study (25). Post discharge data collection for carers was over 6 weeks (with a 3-week post-program follow up period) and care recipient follow up was for 3 months (25). Carers receiving the FECH program in addition to usual care (n=62), compared to those receiving only usual care (n=79) reported:

- Significantly improved preparedness to care from Time 1 (T1, baseline) to Time 2 (T2), approximately 3 weeks later (p=0.006, d=0.52, 95% CI 0.088 to 0.519), a difference sustained at T3, 6 weeks after discharge (p=0.019, d=0.431, 95% CI 0.043 to 0.473).
- A significant reduction of carer strain from T1 to T3 (intervention group mean = -0.15, SD = 0.55; control group mean = 0.04, SD = 0.65; p = 0.040, d = -0.316).
- A significantly greater decrease in carer distress from T1 to T2 (intervention group mean =
  -0.24, SD = 0.46; control group mean = -0.09, SD = 0.64; p = 0.036, d = -0.269) (25).

In addition, hospital usage by care recipients in the follow-up period was (non-significantly) lower for older people whose carers received the FECH program, compared with controls (28). However, this preliminary work was in one setting, the program needed to be extremely brief, the study was only powered to detect changes in caregiving preparedness, and patient and carer follow-up periods were short. The current study will examine impacts from a 6-month version of the FECH program, designed to further embed the problem-solving approach for carers; it will provide more generalisable findings; be powered to detect clinically important changes in carer, care recipient, health system, and cost outcomes; and examine change over substantially longer periods. Both cost utility analyses and effectiveness analyses will examine caregiving sustainability as an outcome.

In summary, a critical challenge facing Australia is the need to ensure that the rapidly increasing population of older people may remain at home for as long as they and their carers desire, while minimising risks to carer health. Meeting this challenge will have substantial benefits for Australian society as a whole. Our study will determine the extent to which the FECH program can help to meet this challenge. Findings will provide robust evidence, which, if outcomes are positive, can underpin changes in practice and policy in metropolitan Australia and inform similar initiatives elsewhere.

Aims and Hypothesis

Aims of this trial are to:

(1) Measure impacts from participation in the FECH program on the HRQoL of carers of older people discharged home from hospital **(primary aim).**

(2) Measure impacts from participation in the FECH program on (a) other carer outcomes (preparedness to care, self-efficacy, strain, distress, use of health services, use of medications), (b) patient outcomes (symptom distress, level of independence, use of health and residential care services, use of medicines), and (c) costs for health services, medicines, and residential care.

(3) Explore and describe how caregiving is influenced by the program.

Hypotheses are that:

(1) *Carers* participating in the FECH program in addition to receiving usual hospital discharge support, when compared with carers receiving usual discharge support alone, will (during the program and for the following 6 months):

   a) report improved HRQoL, - better self-rated physical and/or psycho-social health expressed as improved AQoL-8D scores (29, 30) (**primary hypothesis**);

   b) report improved caregiving preparedness (higher scores on the Preparedness for Caregiving Scale [PCS] (31)), decreased carer strain and distress (lower strain and distress scores on the Family Appraisal of Caregiving Questionnaire-Palliative Care [FACQ] (32)), and improved caregiving self-efficacy (higher scores on the Caregiver Inventory [CGI] (33)); and

   c) experience decreased emergency presentations to hospital, days in hospital, ambulance trips, and use of primary health services/medications for their own health problems (from health system data and carer reports).

(2) The FECH program will be cost effective for carers, defined as having an incremental cost-effectiveness ratio of less than AUD$50,000/QALY gained.

(3) Older people whose carers participate in the FECH program in addition to receiving usual discharge support, when compared with older people whose carers receive usual discharge support alone, will experience (during the program and for the following 6 months):

   a) decreased symptom distress (lower scores on Symptom Assessment Scale (SAS) (34)); improved independence (higher scores on Barthel Activities of Daily Living Index (BADLI) (35)); and

   b) delayed permanent admissions into RACFs (from carer reports), plus fewer emergency presentations to hospital, days in hospital, and ambulance trips, also decreased medication use and use of primary health services (from health system data), resulting in overall decreased costs to the health and residential aged care systems.

Expected Outcomes

Carers support over 2 million older people in Australia (7), helping them to fulfil wishes to live, and ultimately die, at home. As outlined in this proposal, their role is of critical importance, not only to the older people receiving their care, but also to society as a whole. Yet health risks from caregiving can be severe, many carers are older people themselves, and, in findings from a recent survey, over 90% of older carers reported chronic health problems (8). When a carer's health fails, the older person receiving care may experience distressing admissions into hospital and/or residential care. Costs to society are substantial for either eventuality. The FECH program provides a new approach to recognising and supporting these carers of older people, seizing an opportunity to identify them when the older person is hospitalised, elicit their perspective of their caregiving support needs, and empower them with a problem-solving approach to address these needs after the care recipient's discharge and in the longer term.

The FECH program is unique in that it is:

(a) carer led, during contacts facilitated by the FECH Nurse; and

(b) designed to develop the carer's long-term (problem-solving) approach to accessing support.

This approach promotes sustainable caregiving. The approach is also consistent with sustainable health care, in that it supports the required 'connection' between hospital and community care (36).

Project Design

Methodological Approach

The proposed single-blind, multi-centre, RCT (n=648 dyads after estimated attrition of 30%) will evaluate the effect of providing usual discharge support plus the FECH program on carers' HRQoL compared to providing usual discharge support alone. This design, with assessors blinded to the group assignment of study participants, has been chosen to ensure that the strongest possible evidence will be obtained to either support or refute the study hypotheses. The study also directly compares outcomes from usual care to those from usual care plus the FECH program in terms of carer outcomes directly addressed by that program (preparedness to care, carer self-efficacy, carer strain, and carer distress). Further outcomes for carers, care recipients, the health and residential care systems, and related costs, will also be evaluated.

A 1-year follow-up period for carers will facilitate accurate determination of Quality Adjusted Life Years (QALYs), required for cost utility analyses. This timeline also allows for the lag time occurring when using linked data for follow up of carers and care recipients (time for data to enter the 'system' and queuing for data extraction).

Qualitative data from carers will explore and describe carers' experiences of the FECH program in the short and long term, particularly how the program influences caregiving. Process evaluation will be concurrent with FECH program administration.

Settings

Three public hospitals in Queensland (Qld) and WA will participate. In WA, the hospital will be Sir Charles Gairdner Hospital (SCGH), a metropolitan tertiary teaching hospital with approximately 600 beds. In Queensland, the two Hospitals will be Robina Hospital (approximately 400 beds) and Gold Coast University Hospital (approximately 600 beds). Wards with medical patients will be included. Within SCGH, the Discharge Ward (via which area most patients are discharged) will also be involved, in that some patients and their carers will be recruited there. In Gold Coast University Hospital, the Transit Lounge may also be involved in this way.

Control Condition (usual care)

Usual discharge care at all of the included hospitals includes the patient and/or carer receiving:

(a) a copy of the discharge letter,

(b) medications or prescriptions and related advice,

(c) referral letters and/or outpatient appointments,

(d) information about treatments to be carried out at home (e.g., wound dressings), and

(e) information about care packages or programs put in place.

Social work input for carers is not routine but occurs in instances prioritised by the social work department.

Intervention (FECH program + usual care)

The FECH program is a telephone based, post-hospital-discharge, hospital outreach protocol implemented for individual carers by one or more specially prepared FECH nurses with acute gerontological nursing care experience and substantial knowledge on how to navigate the home care 'system' and home caregiving resources. Phone calls (therapeutic contacts) are supplemented with email and mail correspondence, as agreed with the carer, when resources or information are provided by the nurse, rather than accessed directly by the carer from other sources. The program is designed to incorporate identification of the carer by the inter-professional team during the care recipient's hospital admission. However, during the trial, carer identification occurs as part of recruitment.

The FECH program has been enhanced for testing in this study. The program is now to be delivered over 6 months, rather than just a few weeks, adding three additional contacts with the FECH nurse. This lengthened period is to further embed the processes of carers:

(a) reflecting upon the current caregiving situation,

(b) identifying and prioritising new or ongoing support needs, and

(c) implementing a problem-solving approach to address these support needs.

The intention is that carers will then have the problem-solving skills to continue without support from the FECH nurse, eliciting help from organisations such as Carers WA or Qld when needing to access support services in the future.

The FECH program now involves six contacts by the FECH nurse with the carer over the six months after the care recipient's discharge home from hospital (at 1 and 2 weeks after discharge, then at 1, 2, 4, and 6 months post discharge). During Contact 1, the FECH nurse first elicits the carer's understanding of information provided to them, at the time of the discharge, by the hospital. This occurs during a brief telephone interview lasting, on average, 15 minutes. If the carer demonstrates insufficient understanding of discharge information to form the basis for caregiving, as determined by the nurse when this is explored during the

interview, the FECH nurse guides the carer to access the information required. The Carer Support Needs Assessment Tool (CSNAT) (40) (attachment to submission) is provided to the carer, usually by mail, but with an email option, to allow the carer time to reflect upon the included items before Contact 2 occurs. The FECH Program Carer Booklet, which summarises key elements of the FECH program, will also be provided at this time.

The CSNAT could be used as an outcome measure, however, this is not the case in the current study. Instead, it is a key component of the intervention. The CSNAT was first developed for use in palliative care and has 14-items with Likert-type response options that rate needs for support in two domains: enabling the carer to care for the patient at home and enabling support for the carer in their caring role (7 items in each domain). More support options may be added/rated by the carer (40). When the CSNAT was trialled in home based palliative care in WA, carer strain was reduced (41), and the likelihood of achieving the preferred place of death was increased (compared with controls) (42), a finding that tends to support use of the CSNAT as part of a proactive approach to caregiving rather than just one that responds to immediate needs.

At Contact 2, the FECH nurse guides the carer through the reflective CSNAT approach, using the CSNAT to identify and prioritise their support needs, then to devise ways in which up to three prioritised needs will be addressed using problem-solving techniques (9, 40). Contact 2, (first time of using the CSNAT approach and problem-solving) will take 45 minutes on average, based on our preliminary work.

At Contacts 3-6, each taking 30 minutes on average, the carer will be prompted to reflect upon the extent to which support was accessed as planned since the previous contact, then again use the CSNAT approach combined with problem-solving to identify, prioritise, and address remaining or additional support needs. Each contact point provides an opportunity to reinforce the problem-solving skills learnt.

The FECH nurses from WA and Qld will be trained together, in Perth (to ensure consistency and collegial rapport), over 3 days. Training was developed for our preliminary study and is now augmented with sessions on embedding the problem-solving approach and with skills practice. Sessions will address:

(a) determining the extent and type of discharge information provided to the carer, any clarification needed or additional information required (CIs Toye/Slatyer[nurses], A-M Hill[physio]);

(b) using the CSNAT approach (CI Aoun [CSNAT expert]);

(c) embedding the problem-solving approach for carers so that it can be used in the longer term, after the program has ended (CI Bryant [clinical psychologist]); and

(d) types of resources that may be helpful to carers (CI Toye with input from other CIs and AIs).

Training materials include a CSNAT toolkit (43) and an electronic manual guiding access to supports for carers related to care provision for older people in Australia (44). The previous published study, plus some additional recently completed pilot work, will inform scenarios presented to the nurses to allow them to practice and hone their skills. For example, in the more recent work, participants have expressed concerns about how to minimize falls risks and how to support adequate nutrition in older care recipients.

One of the aims of the intervention is to provide carers with problem solving skills that they can continue to use after the end of the intervention period. FECH nurses will emphasise the time-limited nature of their contact, and in each session encourage carers to actively use the skills discussed for identified problems, or the broader problem of future planning. At the last FECH nurse contact, carers and the nurse will review the strategies and resources used, highlighting which were most useful, and refreshing any skills that the carer feels unsure about. Carers will also be provided with a brief summary sheet of the skills they have used and may continue to use (End of FECH Program Summary for Carers).

Participants

Consistent with the definition of a carer used in an Australian study investigating the support of frail older people (37) we regard family members or friends providing unpaid care and support to older people as carers. Included dyads will comprise (a) one adult (aged 18+) carer who provides regular[1], ongoing[2], home-based physical and/or emotional care to a person aged 70 or older, when this person is returning home[3] (discharged) from an included ward, and (b) that older person (the care recipient). In Qld, a person remains a hospital patient when receiving Hospital in the Home and Rehabilitation in the Home. Therefore, the patient and carer cannot be included in the study until discharged from that program. In WA, patients are discharged from hospital before receipt of these programs so can commence study inclusion as soon as they have left the hospital. These hospital services will be documented by the study team via information received from the family.

We will include patients who are unable to consider consenting because of cognitive impairment (including when acute illness is compromising cognition) via a waiver of consent (Western Australia) or consent provided by a substitute decision maker (Queensland). We will also seek to include patients who do not speak or understand English when we are able to provide a translation of the study information sheet and consent form in a language that they understand and we can answer any questions from the patient via a member of the patient's family who can translate for them.

We will ensure that we address concerns regarding vision and hearing. We will address issues of poor sight (eg, by providing documents in large font, good lighting) and poor hearing (eg,

---

[1] At least weekly
[2] Likely to continue for at least 6 months
[3] To their own home or that of a friend or relative

checking that hearing aids are used and working, minimising exposure to background noise, facilitating 'lip-reading', speaking slowly and clearly but refraining from shouting).

We will exclude dyads when patients do not return directly home from hospital (eg, they return home via a rehabilitation setting or an aged care facility); when the carer cannot speak, read, or understand English; or when the carer's hearing prohibits their ability to hold telephone conversations. Testing with other (eg, culturally and linguistically diverse) carer populations is a later goal for our team but will require significant additional resourcing for both the FECH program and outcome evaluation. Therefore, comprehensive evaluation in an English-speaking population is the more immediate priority.

If a carer dies or is admitted into an RACF (for ongoing, ie not respite, care) during the data collection period, data collection from the dyad will cease. If a patient dies or permanently enters a RACF, the carer will be offered the opportunity to withdraw from the study or to continue in the study as before (in the study arm to which they were assigned), with the FECH program tailored to that new situation if they are in the intervention arm. Information about bereavement services will be provided, if appropriate, to carers deciding to either withdraw or continue with the study. In each instance, the provision of such information will be recorded. When carers in the control group are provided with such information, consideration will be given to excluding them from analyses determining the effectiveness of the FECH intervention.

When the following scenarios eventuate, we will document accordingly so that study analyses can take account of them:

1. The unpaid carer who is recruited becomes a paid carer (providing care package type services to the older person).
2. The patient is included in the CoNeCT (complex care coordination) program in WA https://www.fhhs.health.wa.gov.au/Our-services/Service-Directory/CoNeCT-Complex-Needs-Coordination-Team or any equivalent program in Qld.
3. The patient is included in a transition care program (TCP) at home https://www.myagedcare.gov.au/after-hospital-care-transition-care
4. The patient is included in another post hospital discharge program, such as that run by Silver Chain https://www.silverchain.org.au/wa/aged-care-needs/following-a-recent-hospital-stay/
5. The carer is included in the Gold Coast Health Nurse Navigator program.[4]

Power Calculations

The primary outcome is the change in total score on the AQoL-8D (13, 14) for the carer. Hawthorne and Osborne (37) consider that a very small effect size of 0.06 may be of clinical

[4] The GCH Nurse Navigator is an advanced practitioner within an interdisciplinary team who works closely with the patient and their family to provide continuity of care both across acute and community care

importance in relation to this instrument. However, we anticipate a larger effect size, based upon:

(a) our assessment of changes in health, measured using the SF12 (38) during our preliminary study (9), in which we obtained positive change (effect sizes) from baseline until immediately post-intervention in physical health (0.17) and mental health (0.22);

(b) that the AQoL-8D is a more appropriate outcome measure than the SF-12 for this study as psychosocial components of health are emphasised more; and

(c) that we are now implementing an expanded intervention with longer follow up.

Therefore, we designed this study with 80% power to detect an effect size of 0.22. This would require 324 carer dyads in each of the study arms (control and intervention), determined using the G*Power sample size calculator (39).

Recruitment

Our recruitment target is 925 dyads to accommodate anticipated attrition of 30% and achieve the final sample of 648 dyads. Recruitment, baseline assessments, and outcome assessments will be undertaken by a team of Project Officers (POs).

Identifying potential participants. As in our preliminary study, POs will liaise with the ward staff to identify carer-patient dyads that might meet study inclusion criteria. The following approaches will provide information to the staff working in the areas where study recruitment will be undertaken and/or to potential patient and carer study participants:

1. Articles explaining study plans/progress in hospital staff newsletters.
2. Articles explaining the study in external organisations' newsletters provided to carers, such as those distributed by Alzheimer's WA or Carers WA.
3. Oral presentations at staff meetings and Multi-Disciplinary Team (MDT) forums when recruitment is about to commence and when it is finished – with updates as necessary to ensure that all the relevant staff remain aware of the study activity.
4. Information about the study on posters displayed in the participating wards.
5. At SCGH, subject to hospital approval, slides will be uploaded onto the hospital TV to explain the study and provide details of how to contact the study team.
6. Flyers will also be provided to all participating wards. These flyers will briefly describe the study and who might be eligible to take part. The ward staff (nurses, allied health staff, doctors) will be asked to distribute these flyers to patients and/or families that might meet study inclusion criteria, and they will also be available for the research team to hand out when explaining the study. The flyers will have a contact number for queries and a tear off slip that the patient or family member can complete with contact details if they would like to know more about the study. Boxes will be placed on the wards so that these slips

can be dropped into them and the research staff will follow up on requests for information on a daily basis. Subject to the agreement of the hospitals, a flyer will also be placed into each discharge envelope of an older patient returning home, in case they have not yet had an opportunity to learn about the study.

The wording for the flyers, posters, and slides is in submitted with this protocol (see wording for flyers, posters, and slides). All these items will conform to the style requirements of the hospitals/health services in which they will be used.

Subject to agreement from the relevant Head of Department at the hospital, staff members already calling the family for other reasons may mention the study to them if they think the patient-family dyad might meet inclusion criteria. A brief script (submitted as an attachment, 'brief script') will be provided to these staff members that explains the study, provides study contact details, and asks if the carer would like to share their contact details with the research team so that they can be called with further information.

Obtaining consent._Prior to the discharge, study staff (POs) will explain the study to potentially eligible carers and patients who are agreeable to learning more about the trial. At this time, they will provide and explain the study Information Sheets and Consent Forms (PICFs), including a separate form for the Department of Health and Human Services [DHS] (now Services Australia) that is for all participants able to provide consent. A Participant Information Summary will also be provided to both carers and patients along with the relevant PICF.

Although the detail of how this process will be operationalised in each hospital ward may vary, the principles will remain the same in that the ward staff will (a) help to identify potentially eligible patients, (b) introduce study staff to potentially eligible patients, and (c) help to facilitate contact between the study team and the patient's family if the family are agreeable to this. Often, more than one family member or friend might be eligible for recruitment along with the patient, and sometimes the first person may not be eligible but another family member or friend might be. In these instances, the study staff will provide the relevant information to the first family member contacted and request that they might then be put in touch with any other person deemed to be both eligible and more suitable.

When eligible carer and patient participants are willing to take part, they will be asked by the POs to provide either written informed consent, or witnessed and documented verbal agreement to participation if they have a disability that precludes providing a signature on the consent form (see form for witnessed verbal consent). If they need more time to consider consenting, carers will be asked to provide contact details for follow up. When these contact details are not provided directly to the POs, there will be an option to complete a tear off slip at the bottom of the information sheet and place this in a box on the ward requesting contact from the research team. Recruitment conversations with the patients and carers will allow time for their questions to be answered. Additional time to consider the opportunity, and discuss it with others, will be available up until such time that allows baseline data collection

to be completed by approximately one week post-discharge, given that those in the intervention group are designated a FECH contact after that week.

If the dyad has not met with the POs during the admission but expresses an interest in learning more about the study within the days immediately following the discharge (eg, by calling the number provided on the flyer), the information sheet and consent forms will be sent to the carer and patient via mail or email, and including the Participant Information Summaries. Signed consent forms can be returned in pre-paid study envelopes or via email. Documented witnessed verbal consent to study participation (as described before) will be accepted by phone, if face-to-face contact with the research team is not feasible, so that data collection and group assignment may commence without delays such as those incurred via postal services when the potential participant has no access to scan and email documents. However, signed written consent is required at least for accessing Department of Human Services (DHS) – now Services Australia - data and will, therefore, be sought by our team within the first month of study enrolment, except in any instance when the patient is included via a waiver of consent (in WA), or a disability means that a signature cannot be obtained.

For patients, we will endeavor to seek consent at a time when their condition does not interfere with their decision-making capacity related to consideration of study participation. This may mean approaching them in the morning, before any treatment or activity has tired them. If there is some doubt about the patient's cognitive capacity before we approach them – such that they might be able to consider consenting but a medical opinion is necessary to be sure – we will check with the medical team prior to approaching the patient. When we approach the patient and explain the study we will also check back to ensure their understanding before asking for consent. If there is a limitation such that written, as opposed to verbal, consent is compromised, witnessed verbal consent will be accepted with the process documented by the project officer.

Patients who are unable to consider consenting. When a patient is unable to consider consenting because of cognitive impairment, which may relate to acute illness or a long term condition, we will seek to include them in the study if the carer is willing to take part. We seek to include these patients via a waiver of consent (in WA) or (proxy) consent from the patient's substitute decision maker (in Queensland) (see Figure 2).

The following process will be undertaken to identify a substitute decision maker in Queensland:

1. If there is person with enduring power of attorney for personal matters or a guardian appointed by the Queensland Civil and Administrative Tribunal (QCAT) who can be approached in the limited time available to access consent for the patient to be included in this study, this approach will be made.
2. If such a person is not available, the statutory health attorney (as defined in the Powers of Attorney Act 1998 (Qld)) will be approached. Such a statutory health

attorney is the first of the following people who is readily available and culturally appropriate to consider this issue:

- The husband or wife of the patient (if the relationship is current and close)
- An adult (unpaid) carer of the patient
- An adult who is a close friend or relation of the patient and is not their paid carer

Since the adult, unpaid carer of the patient is the other member of the dyad to be recruited for this study, it is not foreseeable that any patient in Queensland would need to be included via a waiver rather than via consent obtained from a substitute decision maker.

If the patient is included via a waiver of consent (WA) or proxy consent (Queensland), an opt out form for the patient to complete later will be provided to the carer (see Opt Out Form). Then, if the patient recovers sufficiently to do this, and prefers to withdraw, this form is to be provided to the patient by the carer so that the patient can complete and return it in the reply-paid envelope that will also be provided. Withdrawal of consent (ie, opting out) will be possible up until the time when patient administrative data have been accessed by our team.

Figure 2 Recruitment decisions

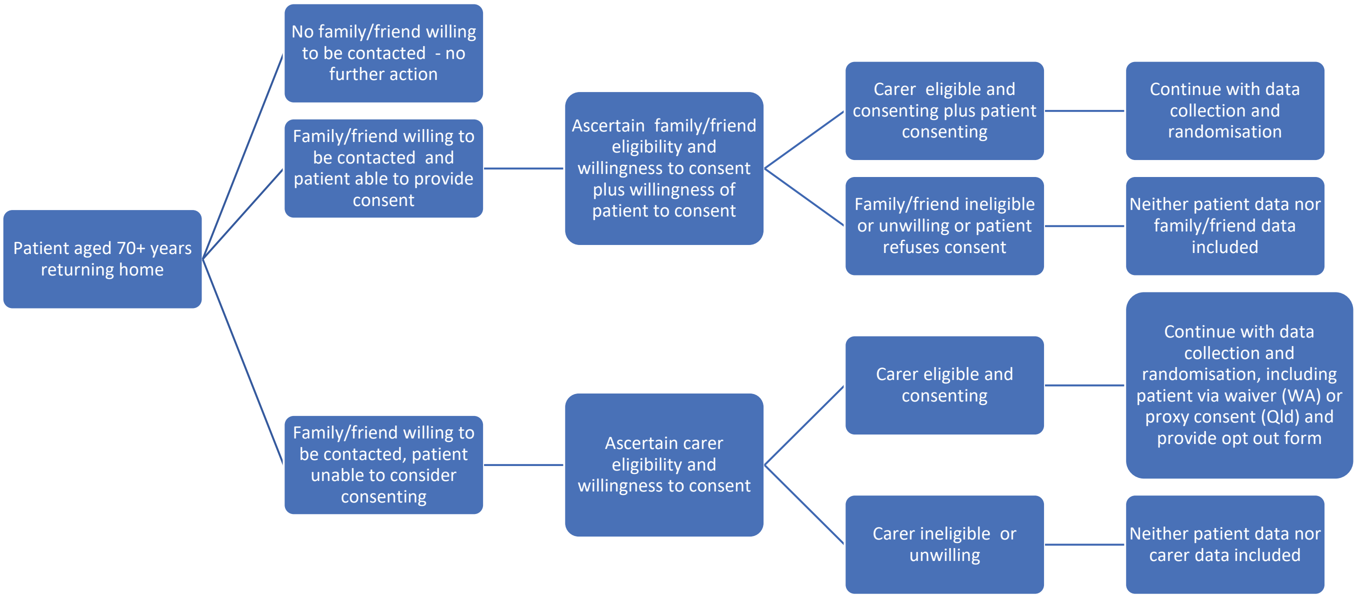

A purposive sub-sample of an estimated 25 carers from WA and Qld who have completed participation in the FECH program will be selected immediately after program conclusion for inclusion in qualitative interviews on the basis of questionnaire data to ensure maximum variation (e.g., carer gender, age, relationship, caregiving duration), until data saturation (see Interview Questions). After an additional 6 months, these people will be asked to participate in a second interview. In all, we anticipate that 40 interviews (allowing for attrition of 10 carers) will be completed in this time frame.

Database Setup, Random Assignment, and Blinding

The Clinical Trials and Data Management Centre (CTDMC) at Curtin University will manage database set up and randomisation processes to ensure the integrity of these processes and of blinding. We will use stratified randomisation so that numbers of participating dyads in the control and intervention groups will be balanced within each hospital. A list of treatment allocations will be prepared before the study; it will contain a study number (whole number, starting from one), and a code to indicate the group (intervention or control). The list will be generated by the CTDMC using computer-generated random numbers, organised so that recruitment to the two study arms occurs at an approximately equal rate. This will be done using a blocking strategy, with half of the numbers within each block allocated to each treatment. Block length will be randomly selected and comprise an even number (from 6-12). This means that, at the end of each block, there will be an even number of dyads allocated to each group (control/intervention). A list of case numbers will be provided to each hospital so that the next dyad to be recruited within the hospital can be assigned the number at that time.

The treatment allocation list will be stored as a password protected file on R drive at Curtin University and will only be accessible to the trial's CTDMC REDCap administrators and any investigators or study staff specifically designated as requiring access for study oversight purposes. The executive officer from the CTDMC will be responsible for setting up this folder and will need to approve access for anyone else. To maintain blinding to group allocation, no investigators or study staff who are involved in recruitment, obtaining baseline data, obtaining outcome data, or analysis of these data will be allowed access.

After a dyad has been screened by the recruiting study staff member and has provided consent to take part in the study, they will be assigned their (matching) case numbers (ie, the case number will only differ in that it indicates whether they are a carer or a patient). Then, after the discharge from hospital, baseline (Time 1 [T1]) assessment will be completed and the PO completing this assessment (or completing the demographic and caregiving details and ensuring that the participant completes the remaining questionnaires online) will inform the relevant State Project Manager (or their deputy) who will trigger the release, to themselves (ie, the State Project Manager), of the group allocation via REDCap. The FECH Nurse will then be notified by the State Project Manager and/or via REDCap to commence the intervention if the dyad is assigned to the intervention group.

The contact details of the carer assigned to the FECH program will be provided to the FECH Nurse by the State Project Manager or their nominated deputy (a study investigator in the same state[5]). In a separate communication, the demographic and caregiving details of the dyad, collected at T1, only identified by the study code, will also be provided to the FECH Nurse by the State Project Manager or their nominated deputy (to avoid asking the carer for information already provided).

Time 2, 3, and 4 data collection will always be prompted by the State Project Manager or their deputy. This strategy is so that FECH program completion occurs prior to T3 data collection (intervention group) and will maintain the blinding to group allocation of the POs. When a previously recruited dyad comes before the recruiting staff on a subsequent occasion (following care recipient re-admission), re-recruitment will not occur but follow-up will continue as planned from initial recruitment.

Treatment allocation will be concealed from the POs, the hospital staff, and any investigators or study staff involved directly in recruitment, trial-related data collection, or data analysis. POs will be located away from the FECH nurses so that no inadvertent 'unblinding' of these POs can occur via face-to-face contact. Similarly, FECH nurses and POs will have separate group email addresses so that inadvertent 'unblinding' via email becomes less likely. In addition, all study participants will be asked by the POs not to mention if study personnel have been in contact with them to ensure they do not reveal their group allocation (saying, 'Before we begin could you please remember not to mention whether or not other study personnel have been in contact with you?').

If, for any reason, a PO becomes 'unblinded' to the status of any particular participant, further trial-related data collection from that person will be undertaken by a different PO (who is not unblinded). Study staff members or investigators involved directly with recruitment for qualitative interviews or the collection and/or analysis of the qualitative interview data from FECH program participants cannot be blinded so will not be directly involved with recruitment, data collection, or data analysis for the trial itself. State Project Managers will have no direct role in recruitment or data collection.

Outcome Measures and Demographic and Caregiving Details

Questionnaires (total 94 items) have been selected to minimise burden yet obtain the necessary data using scales with good psychometric properties in similar populations. These questionnaires are attached.

Demographic, caregiving, and service use details will be captured for carers and care recipients from the carer (see Demographic, Caregiving, and Service Use Details Form). Demographic and caregiving details are accessed at baseline. Other information collected at this time concerns the type, duration, and amount of care provided by the carer and carer/care recipient health. Health and care service use (carer and care recipient) will be reported by carers at all data collection time points (for those completing questionnaires online, these questions will be asked during the phone reminders for online completion), complementing system data.

---

[5] One who does not need to be blinded because they will be involved in any way with data collection or quantitative or economic analyses

Carers' health related quality of life (primary outcome) will be documented using the 35-item AQoL-8D (29, 30) a Multi-Attribute Utility Instrument (MAUI), required for the cost utility analysis, with good psychometric properties, that captures psycho-social as well as physical health domains (Independent Living, Happiness, Mental Health, Coping, Relationships, Self-Worth, Pain, Senses) (30). Australian population norms have been established (38). The focus on psychosocial domains makes this instrument appropriate for our study since the dominant factor affecting choice of a MAUI is its ability to capture facets of health states relevant to the research question.

Carers' self-rated preparedness for caregiving will be measured with the Preparedness for Caregiving Scale (PCS) (31). This 8-item scale has 5 response options (0=not at all prepared, 4=very well prepared) and is designed for use with carers of older people receiving homecare/experiencing care transitions. Cronbach's alpha coefficients were 0.88-0.93, with construct validity established, in older people (39). Testing in patients with life threatening illness confirmed satisfactory internal consistency, reliability and stability, and unidimensionality (40).

Carers' self-efficacy will be measured with the 21-item Caregiver Inventory [CGI] (33)). This questionnaire has four sub-scales confirmed by factor analysis: Managing Medical Information, Caring for the Care Recipient, Caring for Oneself, Managing Difficult Interactions and Emotions. Cronbach's alpha for the scale was 0.91 in a sample of carers of patients for whom the main diagnoses were cancer, chronic obstructive pulmonary disease, stroke, chronic heart failure, and dementia. Responses are provided on a Likert type scale (33).

Carers' caregiving strain and caregiving distress will be measured by the corresponding sub-scales (8 and 5 items respectively) of the Family Appraisal of Caregiving Questionnaire- Palliative Care (FACQ), for which good internal consistency reliability and construct validity are confirmed (32). Responses are on a 5-option scale (Strongly agree-Strongly disagree).

Care recipient independence will be reported by carers using the Barthel Activities of Daily Living Index (BADLI), which has good psychometric properties and evaluates 10 activities (35)

Care recipients' symptoms will be rated using the Symptom Assessment Scale (SAS) (34). Seven symptoms are each scored from 0 (not at all) to 10 (worst possible). Scores are totalled. Proxy responses will be completed by carer proxies if care recipients are too unwell to respond or cognitive impairment precludes their ability to do this, or if the process of seeking symptom ratings might cause distress (as noted by the carer or as indicated in any other way). Further, any indication of impending distress once the rating of symptoms by the care recipient has commenced will trigger cessation of this process (polite withdrawal, for example saying "I can tell that you are a little tired so we won't carry on with this today but thank you for speaking with me"), with caregiver (proxy) ratings accepted instead. Adequate internal consistency reliability and good test-retest reliability was documented in acutely unwell (predominantly older) patients (34). Concurrent validity was demonstrated in an aged care setting (41).

Data Collection or Gathering

Questionnaires (submitted with this application) will primarily be administered by phone by the POs, using consistent introductory scripts (Questionnaire Scripts) that have been piloted and

administering the questionnaires in a consistent, predetermined order. These POs will enter the responses into REDCap via a computer terminal, laptop, or tablet that has a wifi connection. Hard copies (identified by study code only) will be used if REDcap cannot be accessed at the required time and the data from these will be entered into the REDCap system as soon as is feasible after access is regained, after which the hard copy will be shredded. In the interim, the hard copy will be kept securely and separately from the study master list. All responses will be from carers except when care recipients are able to rate their own symptoms (preferred).

The (piloted) option for web-based questionnaire completion by carers, using questionnaire scripts reworded so that they make sense in that context (eg, instead of saying 'please tell me which response', 'please click on the correct response') will be supplemented with reminder phone calls provided by POs. Also, when carers elect to contribute data via the web-based questionnaire, we will offer to support them on the phone if they select this option, the first time they do this. Demographic and Caregiving Details will all be collected by phone to help to develop and sustain the relationship between the participant and these study staff and initial Service Use Questions will be asked when those demographic details are being collected.

From preliminary work, the mean time taken for each questionnaire data collection time point will be 30 minutes (by phone), including time for introductions, establishing any care recipient admissions into an RACF, and reporting use of health services, care services, or RACFs not captured in administrative data. Collecting demographic data at T1 will add 30 minutes. (Table 1).

Table 1. Timing of data collection contacts and FECH contacts (if applicable)*

Contact purpose	*Time Post Discharge*	*Questionnaires used, also: demographics (DD); services used (SU, includes RACF admission); qualitative interview (QI)*
T1 (baseline) data collection**	1-4 days	DD, AQoL-8D, CGI, PCS, FACQ, SAS, BADLI, SU
FECH Contacts 1-4: one week, 2 weeks, 1 month, and 2 months post-discharge		
T2 data collection	3 months	AQoL-8D, PCS, FACQ, SAS, BADLI, SU
FECH Contact 5: 4 months post discharge		
FECH Contact 6: 6 months post discharge		
T3 data collection	6 months***	AQoL-8D, CGI, PCS, FACQ, SAS, BADLI, SU, QI (subset)
T4 data collection	12 months	AQoL-8D, CGI, PCS, FACQ, SAS, BADLI, SU, QI (subset)

* Tool name abbreviations shown in text above. **Followed by random assignment to group.

***After FECH Contact 6 if in intervention group.

Qualitative, digital, audio recorded and transcribed semi-structured telephone interviews (estimated 10 minutes) will be scheduled from Perth (to participants in Qld and WA) at two time points (at the end of program and 6 months later) to explore how the FECH program has influenced caregiving and carer experiences during and after the program.

Research Activities and Timeline

Participants will be asked to commit to follow-up over 12 months. Based upon our previous work in this area, we will recruit 925 patient-carer dyads in total within approximately 18 months (52/month across three hospitals), however, we allow for recruitment to take up to 23 months in the timeline provided for this submission. We will recruit over 18 months and collect data from carers and care recipients over 30 months (see Table 2). Prior to the date when data analysis commences, participants will be able to contact the research team to review their questionnaire and/or interview data, as outlined in the PICFs.

Table 2. Study timeline

Quarters shown (m=months)	**Year 1**				**Year 2**				**Year 3**				**Year 4**			
Recruitment (up to 23 m)	■	■	■	■	■	■	■	■								
FECH program (up to 29 m)	■	■	■	■	■	■	■	■	■	■						
Data collection from caregivers/patients (up to 36 m)	■	■	■	■	■	■	■	■	■	■	■	■				
Finalising data collection/records, data analysis (questionnaire data), receipt of linked data (9 m)												■	■	■		
Data analysis/reporting/dissemination (15 m)												■	■	■	■	■

Impact of and Response to Participant Withdrawal

All those withdrawing from the study will be invited to provide their reason(s) for doing so and these reasons will be recorded. In our earlier study, in which direct data collection was only from carers, attrition was 19.5% in the intervention group versus 8% in controls. In the proposed study, we will also collect symptom data directly from care recipients and the longer follow up is likely to lead to increased attrition from death and health problems. However, if care recipients can no longer respond to questionnaires because of failing health or cognition, proxy reports of care recipients' symptoms will be obtained from carers, which is a recognised strategy for the use of the SAS (18), and access to administrative data will still be achievable.

We anticipate 30% attrition during the 12 month post-discharge period so our recruitment target to address the primary outcome variable is 925 dyads. Based upon preliminary work, this sample size will allow 80% power to detect meaningful differences for carer preparedness, carer strain and distress, and hospitalisation costs for patients (9). In our previous study, we recruited, on average, 7 dyads per week from the single participating 36-bed MAU. A conservative estimate, also based upon data from the additional wards to be included in this study, is that 8 dyads per week will be recruited in WA plus an additional 4 per week from Qld (where discharge home of older patients average 8/week).

Process Evaluation

Process evaluation of FECH program implementation will address fidelity, safety, reach, and dose (42). Data will be collected by the FECH Nurses and recorded in hard copy then entered into an electronic database via REDCap (see FECH Nurse Process Log). Each nurse will document:

(a) adherence to or deviation from planned FECH processes:

(b) information provided to carers and the resources to which they were referred,

(c) the extent to which carers engaged with resources (numbers and types of resources, how and when accessed, with what result [as planned or not and if not, why]);

(d) any safety concerns identified by the FECH nurse and how these were managed; and

(e) time taken to implement processes.

FECH Nurses will be asked to record their reflections on each FECH Program Contact in REDCap as explained to them during the FECH Nurse training, subject to them providing written informed consent to contributing these data (see FECH Nurse PICF). Nurses are asked to consider how well the contact went and why, for example, if explaining the purpose of the contact was problematic or easy, and why this was so. Nurses are also asked to provide any other thoughts on the process that will support the evaluation of the FECH program. Demographic data will be collected from these study participants (see FECH Nurse Demographic Details Form). Contextual factors that may be barriers to, or facilitators of, the effectiveness of the FECH program will also be recorded in the database.

The extent to which implementation occurs as planned (fidelity) will be monitored, with steps taken by the investigators to ensure that this is maintained as soon as any indication of likely deviation is noted. To ensure this, one of the FECH program telephone calls will be recorded for each of the carers assigned to the intervention group (with their consent [see PICF]). When written consent to the recoding has been provided, the FECH Nurse will check (on the phone) that this is acceptable to the carer immediately before any recording is made. The State Project Managers will ensure that recordings are made of each of the 6 FECH contacts in approximately even numbers. Every 3 months during the FECH implementation period, a random audit and review of 5% of these recordings will be undertaken by one investigator, with feedback provided to the relevant State Project Manager and FECH nurses. In addition, (a) the State Project Manager will meet with the FECH Nurses on at least a fortnightly basis during the program to ascertain and address any threats to fidelity and (b) each State Project Manager will let the other know whenever a threat to fidelity has been determined in their state so that the action taken to address this threat is addressed in a similar manner in both states (as is appropriate).

The extent of support accessed (dose) will be determined in two ways:

(a) time spent during FECH contacts and

(b) the extent to which identified needs are met as planned.

Program reach will be established by comparing characteristics of those dyads assigned to receive the FECH program and continuing until program completion with characteristics of 'non-completers', taking into consideration reasons for withdrawal. Descriptive statistics will summarise findings.

Data Linkage

Linked administrative health data from each State's data linkage services will be used to capture health service use for all carers and care recipients for 12 months prior (pre-baseline) and at least 12 months post recruitment (follow up), including: Emergency Department (ED) attendance, admission to hospital, length of stay, and use of ambulance services. Use of services covered by the Medicare Benefit Scheme (MBS) (eg, GP visits) and Pharmaceutical Benefits Scheme (PBS) (medication usage) will be captured via Commonwealth departments. MBS/PBS data will be elicited via the DHS (now Services Australia) for patients providing written informed consent to access these data.

Pre-baseline resource use data will provide additional baseline variables to control for potential confounding or effect modification in the effectiveness and cost effectiveness analyses. The difference between pre- and post-baseline use of health services will be used to measure changes in carers' and care recipients' levels of utilisation and cost of health services for participants in both study arms.

Charlson's Co-morbidity Index (43) will be referred to to determine comorbidity at recruitment for carers and care recipients, using hospital data (when available) supplemented by carer reports. Additional information provided by carers about health and care services received by patients and carers will clarify services used and obtain a more comprehensive picture.

Ethical Issues

Our team has made special provision for the following foreseeable contingencies based upon our previous experiences in similar and related studies:

1. POs, qualitative interviewers, and FECH nurses will be trained to manage the following situations (also to report these situations to the State Manager or their deputy):

- Should carers show any signs of distress during data collection contacts, POs will offer an opportunity to cease data collection at that time, recommencing at a time that is suitable for the participant, and checking to see that they have a plan to access additional support in the interim if needed (eg, from another family member). In exceptional situations (ie, severe distress), the State Project Manager may intervene by checking the group assignment of the participant and contacting the FECH Nurse supporting the participant in the first instance (if applicable), who can then address the situation with a deeper understanding of the carer's situation, otherwise directly advising access to resources such as Carers WA, Lifeline, or similar, based upon their assessment of the situation. The State Project Manager or their deputy would also provide a follow up phone call to check on the status of the participant and whether or not further action is needed.

- Should carers exhibit anger or frustration during data collection, POs will allow the participant time to vent before continuing, and validate what is said. Overall, we will seek to provide continuity in terms of the same POs contacting the same participants at each time point, meaning that the PO builds rapport with the participant and will build some understanding of the context of caregiving based upon what has been said before.

- Should patients report severe symptom distress when reporting their symptoms, POs will ask if this information may be shared with the carer so that action can be taken accordingly. POs will then report this situation to the State Project Manager or their deputy who will (a) inform the carer, (b) inform the FECH Nurse if the carer is in the intervention group, (c) follow up within 24 hours to ensure that the situation has been addressed, and (d) document the scenario and its outcome.

2. FECH nurses and POs will be appointed with the understanding that there needs to be some flexibility regarding the hours that they are available to work, given that carers have multiple commitments. However, their availability will also have pre-set boundaries made known to the carers. Should carers receiving the FECH program need support outside of these hours, it will be a part of the FECH nurse's role to work with the carer to find other sources of such support.

3. All the study staff, but especially the FECH nurses, will be debriefed by the State Project Managers regularly. In previous work, we have found that the situations carers are in, when relayed by carers to the study staff, may be confronting and distressing to these staff members. Therefore, the staff members need an opportunity to talk through their responses without breaking the confidentiality of study participants. State Project Managers will consult with the CI team if they need further support themselves to manage these situations. FECH nurses will also support each other both in virtual forums and state based face-to-face forums. CI CB (clinical psychologist) will be involved in the virtual forums.

4. In our preliminary work, there has been no indication at all that a person refusing to take part in a FECH trial, or withdrawing from it, is at any risk of negative consequences related to their relationship with the clinical staff in the hospital. As per our usual practice, we will not disclose any information about who has declined involvement in the study, or has withdrawn from it, to any clinicians (including those on the study team) or beyond the study team. We will keep a record of who has been approached and who has declined or consented to participate in the study so that potential participants are not approached multiple times, however access to these data will be restricted to those involved in recruitment and data collection, who will have signed declarations of confidentiality and will have been trained for their roles. Our presentations about the study to clinicians will also explain the rights of patients and their carers to decline to take part in the study or to withdraw from it.

5. It is feasible that a patient (care recipient) might decline consent to study participation, or withdraw, when the carer wishes to take part in the study or continue their participation. Our training of project officers will equip them to explain to potential study participants the rights of each individual within the dyad to decline participation so that they can help to ensure that no pressure is put upon patients to participate by carers. If a patient (care recipient) decides to withdraw from the study (or opt out) while the carer would prefer to continue, the State Project Manager will review the situation with the relevant project officer, checking to ascertaining whether or not there are particular barriers to participating that could be overcome (for example, by re-explaining study participation or responding to further questions). When any such barriers have been addressed and the patient still wishes to withdraw, a conversation with the carer will ensure that they are thanked for their input and offered a planned program of withdrawal from the FECH program (if they are included in that intervention) tailored in consultation with the CI who is overseeing the program. Information about alternative forms of support will be made available.

Data Management

In this project, data management will be overseen by a Data Management Committee comprising a representative from the CTDMC, the WA and Qld State Managers, and CI Hill. AI Jones may also join this group. The Committee will set up a process for monitoring and auditing the data and guide all data management.

Figure 3 illustrates the sources and types of data to be collected during this project. As indicated in Table 3, *data security* is primarily addressed by the use of secure data entry via REDCap, secure data storage, and secure data transfer. The *forms in which these data will be stored* are also shown in this table, as are the *purposes for which the data or information will be used and/or disclosed.* Conditions under which access to the data or information may be granted to others *(sharing)*, are listed.

Permission is sought from the ethics committees to waive the requirement for consent to access administrative data related to patient use of health services when the patients recruited in WA are unable to consider consenting, as shown in earlier sections of the proposal. In WA, legal review for the waiver is a step undertaken after HREC approval is obtained and is initiated by the reviewing ethics committee if appropriate.

Figure 3. Sources and types of data

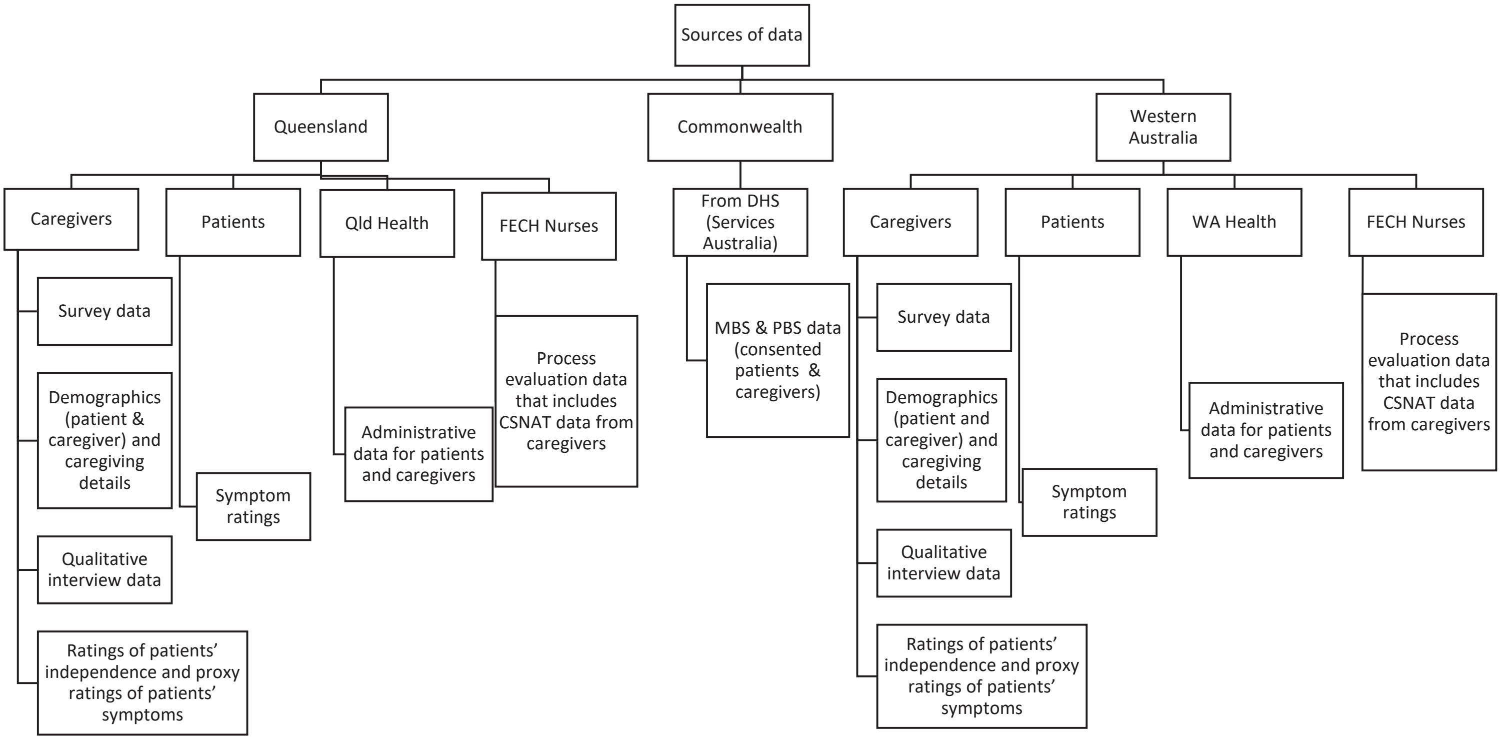

Table 3. Data Management

Source	Generation/collection	Access	Use/analysis	Disclosure/Security	Retention/storage	Disposal	Sharing
Qld & WA **carers** (questionnaire data)	Demographics (patient & carer) and caregiving details. Collected by phone by the research team and entered into REDCap.<br><br>Survey data, collected by phone by the research team and entered into REDCap or directly into REDCap by participant.<br><br>Ratings of patients' independence and proxy ratings of patients' symptoms Collected as per survey data.<br><br>If REDCap cannot be accessed when needed at any time, the data will be entered into a hard copy version identified only by the study code and shredded as soon as access to REDCap has been regained and the data from the hard copy entered into it.	Data accessed via REDCap held securely by the CTDMC at Curtin University. Access during the data collection period restricted to REDCap administrators from the CTDMC and the study's Data Management Team.<br><br>After data collection is completed, the data will be exported into a file ready for analysis.<br><br>Before administrative data are accessed for merging with the questionnaire data REDCap study entries (which include names and dates of birth) will be 'locked down', so that they can only be accessible to the REDCap administrators and persons authorized to check the data (eg, HREC representatives).	Used to establish differences in outcomes between the intervention and control groups.	Master lists held separately and securely (not in REDCap but at the hospitals where the participants were recruited. Sent to the data linkage services when questionnaire data collection is complete.<br><br>Exported questionnaire data identified only by a code, not by name. Shows an indication of group assignment (eg, Group A or Group B) but not what that means (so the analysis for effectiveness can be blinded).<br><br>The treatment allocation list will be stored securely and separately on the research drive at Curtin University.	After questionnaire data collection has been completed, the exported database will be stored on the secure research drive at Curtin University.<br><br>Any hard copy data will be stored in a locked cupboard in the Nursing and Midwifery Research and Education Centre at GCH (Qld data) or the Centre for Nursing Research at SCGH (WA data).	Electronic data deleted 25 years after the publication of study findings.<br><br>Hard copy surveys shredded when the data have been entered into REDCap (see column 1).	Data collected in Qld via REDCap will be held in WA.<br><br>Any electronic data not in REDCap will be transferred as necessary for analysis and storage via the secure upload and download systems of Curtin and Griffith Universities so that all data transfers are secure.
Qld & WA **carers**	Qualitative interview data collected by phone from carers in both	Interviewers plus the investigators designated to oversee	Used to describe experiences	Electronic transcripts identified only by	Interviewers plus the investigators designated to	Used to describe experiences of the FECH	Electronic transcripts identified only

Source	Generation/collection	Access	Use/analysis	Disclosure/Security	Retention/storage	Disposal	Sharing
(interview data)	states (WA interviewers), digitally audio recorded and transcribed.	and be involved with analysis, transcriptionist.	of the FECH program using thematic analysis.	study identifier (code).	oversee and be involved with analysis, transcriptionist.	program using thematic analysis.	by study identifier (code).
Qld & WA **patients**	Symptom ratings collected by phone from patients as per carer proxy ratings of patients' symptoms.	As per carers' proxy ratings of patients' symptoms					
Qld & WA **Data Linkage Services & DHS (now Services Australia)**	Administrative data for patients and carers in obtained from the Qld Health Data Linkage Services (DLS, Qld participants) WA Health Data Linkage Branch (DLB, WA participants) and the DHS (MBS and PBS data). All these data will be merged with the questionnaire data (identified only by study code), Medicare numbers having been replaced with study codes for the DHS data.	Only those investigators and staff who are involved in the analysis process will have access to these data. These individuals will have had no access to the study master lists that provide the identifying personal information for study participants to the DLS and DLB.	Used for effectiveness analysis related to use of health services etc. Then used for the economic analyses.	After checking that any queries about linkage and matching are resolved, the data will be de-identified before analysis by destroying the study master lists.	Electronic file stored on the secure research drive at Curtin University in a project specific password protected folder. Access to the folder restricted to researchers involved in the project who have signed the appropriate confidentiality agreement forms and who have been granted access by the CPI.	Deleted after 7 years from publication.	Participant details provided by the research staff to data linkage services. DHS consent forms scanned and uploaded to the secure research drive at Curtin University and provided to the DHS via secure means.
FECH Nurses in Qld & WA	Process evaluation data entered into REDCap by FECH nurses: (a) adherence to or deviation from planned FECH processes	As per carer questionnaire data.	To help determine the fidelity, safety, reach, and	Exported data identified only by a code, not by name.	After data collection has been completed, the exported database will be stored on the secure	Deleted after 25 years from publication.	Data collected in Qld via REDCap will be held in WA.

Source	Generation/collection	Access	Use/analysis	Disclosure/Security	Retention/storage	Disposal	Sharing
	(b) information provided to carers and the resources to which they were referred,<br>(c) the extent to which carers engaged with resources<br>(d) any safety concerns identified by the FECH nurse and how these were managed; and<br>(e) time taken to implement processes.<br><br>Also recorded will be contextual factors that may be barriers to, or facilitators of, the effectiveness of the FECH program.		dose of the intervention.		research drive at Curtin University.		Any electronic data not in REDCap will be transferred as necessary for analysis and storage via the secure upload and download systems of Curtin and Griffith Universities so that all data transfers are secure.

The following paragraphs complete our data management plan.

The relevant *policies and procedures* taken into account when developing our data management plans include those of the health services, Universities, and the DHS (now Services Australia). Importantly, University regulations dictate data storage for 25 years from the publication of results from a clinical trial. However, health department data and qualitative data are required to be destroyed in accordance with the NHMRC guideline of 7 years from the publication of results.

When considering *contractual and licensing arrangements and confidentiality agreements,* we recognise the agreements that are often required for the use of questionnaires/tools (including the CSNAT tool). Agreements will be put in place, as required, by the Research Office at Curtin (ROC) and/or Griffith University (as required). Confidentiality agreements are entered into by all investigators and research staff as they join the project, via the Research Governance System of WA Health.

Project staff will all undergo *training* for their roles in the project, as appropriate. For example, the FECH Nurses will all undertake a three-day training course in Perth prior to commencing their roles. The research staff employed to recruit study participants and collect data (POs) will be provided with the necessary training by the relevant state-based investigators. Any new employees replacing others during the study will also be trained as appropriate.

Information from the data management plan that needs to be communicated to potential participants includes what data they will be asked to contribute and how, what data about them will be accessed from elsewhere, and what will happen to these data (including how it will be shared, stored, used, and destroyed). Therefore, these issues are explained in our study information sheets.

Risk management has been carefully considered as we have developed our data management plans informed by NHMRC guidelines. Additional steps, not previously detailed, include:

(a) reducing the risk of error via frequent checks of data entry conducted by the State Project Managers;

(b) applications for data approved by custodians of the administrative data that ensure these data, when used by the researchers for data analyses, are non-identifiable (following principles of separation of identifiable linkage data and non-identifiable service data); and

(c) the completion of project staff training in the research module addressing good clinical practice whenever these staff are involved with recruitment, data collection, or data management.

<u>Regarding data storage</u>, the data sets containing de-identified administrative data will reside only on a password protected drive (the secure Research Drive at Curtin University) in a project specific password protected folder. Access to this folder will be restricted to researchers involved in the project who are subject to the appropriate confidentiality agreement and have been granted access by the CPI. Access to the research drive is limited to Curtin University staff and research students and data on this drive are protected via the Curtin firewall, antivirus software, and automatic backups. All computers used for access to these data will have the screen lock set at 5 minutes of inactivity and will be password protected.

After data collection has ceased, all remaining data sets will be stored in a separate folder on the same secure research drive. Access to this folder will also be restricted to researchers involved in the project who are subject to the appropriate confidentiality agreement and who have been granted access by the CPI. However, access may be made available to additional research staff to those eligible to access the administrative data.

During data collection, the data collected via REDCap will be stored in the secure CTDMC environment (see Table 3 for access details). *Table 3 also addresses data archiving and destruction.*

Statistical Analysis

Statistical analysis for effectiveness will be led by CI Parsons and AI Bulsara, biostatisticians. In our preliminary work, there was <20% missing data. Because it is difficult to establish if data are missing completely at random (MCAR), partially missing at random (MAR), or not MAR, we will use Multiple Imputation (MI) methods informed by a sensitivity analysis to manage this, creating 25 or more data sets. Two analyses will be performed, namely: an analysis using only the observed data, and secondly, after missing value substitution, where necessary. Data will be analysed using an 'intention-to-treat' (ITT) approach. Secondary outcomes will be analysed in a similar manner to the primary outcome. Statistical analyses will be conducted using SAS version 9.2 software and a p-value <0.05 will be taken to indicate a statistically significant associations/differences in all tests.

Descriptive statistics will summarise the profile of study participants. These will be presented as frequencies and percentages for categorical variables; means, standard deviations, medians and ranges for variables measured on a continuous scale. Differences in these demographic and baseline health status variables between groups at baseline will be compared using Chi-square, t-tests or non-parametric Wilcoxon 2-sample tests as appropriate. Changes from baseline in the AQoL-8D score for the carer will be calculated to each time point and tested for normality using the Shapiro-Wilk statistic; if not normally distributed, a Box-Cox transformation will be applied to the measure before further analysis. Comparison of the changes in AQoL-8D scores between control and intervention groups will be performed using a mixed model (regression model with the carer group identified as a random effect). This model takes into account correlation between repeated measurements on each individual. An interaction term between time and group will be introduced into the model to test whether rates of change in the outcome differ between groups. If differences between the groups are evident at baseline, these will be included in the model as covariates so that adjustment can be made before examining differences between groups in outcomes.

Preparation for economic analyses. As noted above, carer health state utilities will be captured at baseline and for the previous 12 months for all carer participants who meet the inclusion criteria, and at 3, 6, and 12 months post recruitment. The AQoL-8D (13, 14) utility algorithm weighted for the Australian population will be used to derive an overall index of the health state utility at each time point. The QALY profile for each carer will be calculated using area under the curve methods.

Cost utility and cost effectiveness analyses. These analyses will be led by CI Moorin, a highly experienced health economist and will evaluate all outcomes that show a significant difference across the arms of the trial. Cost effectiveness of the intervention will be measured using the

framework of a within trial cost utility analysis. An incremental cost-utility analysis will be undertaken to compare the mean incremental cost and quality adjusted life year (QALY) profiles for each group according to intervention status. We will also estimate cost effectiveness utilising measurement of the change in other secondary outcomes where a significant difference is observed between the intervention and control groups. Confidence intervals will be presented around the incremental cost effectiveness ratios (ICER), and cost effectiveness acceptability curves for varying threshold values of cost effectiveness will be presented. A 12-month time horizon will be used and a health system perspective using within trial probabilities and costs will be undertaken. Assessment of the sensitivity of the results obtained to variation in measured effectiveness, health care resource use, intervention and usual care unit costs and participant subgroups will be undertaken using one-way and probabilistic sensitivity analysis, as per best practice guidelines (51).

Qualitative Analysis

Thematic data analysis will be undertaken independently by two suitably experienced persons, led by CI Slatyer (expert in this area). Strategies to enhance trustworthiness of findings will include verbatim transcriptions of audio-recorded interviews and a clear audit trail (52).

Results, Outcomes and Future Plans

This proposal was developed with carer input supported by Carers WA and the Consumer and Community Health Research Network, helping to ensure the appropriateness of the intervention and study processes. Consumer advocates continue to inform the study, which is grounded in robust preliminary work and designed to provide valid results to inform practice. As the study gets under way, we will appoint an advisory group that who can guide our external communication and dissemination plans. Important links will include with State and Federal Governments and others involved in health policy and planning, such as the WA Health Services Boards, primary care networks, etc. We will provide a 6 monthly newsletter on study progress to the study team, the participating hospitals, and other stakeholders identified as appropriate by the advisory group. To disseminate findings and ensure that those involved are aware of study closure, a final report will be produced, a media release will be circulated, and Carers WA and Qld will be asked to publish information about study closure and how to access findings on their website. Papers will be published in leading journals, and practitioner and consumer forums will be held in participating hospitals. Study participants will also be provided with a summary of study findings upon request. Dissemination strategies will all be logged. There is no anticipated secondary use of data at present but follow-up studies may be proposed, depending upon results.

References

1. AIHW. Life expectancy and disability in Australia: Expected years living with and without disability. 2017.
2. AIHW. Older Australia at a glance. 2016.
3. AIHW. Australia's health 2016. 2016.
4. COTA Australia. Summary report on the conversations on ageing. 2012.
5. Productivity Commission. Introducing competition and informed user choice into human services, draft report. 2017.
6. Carers NSW. Carers NSW 2016 Survey. 2016.
7. Australian Bureau of Statistics. Disability, ageing and carers, Australia: 2015-2016. 2017.
8. Jowsey T, McRae I, Gillespie J, Banfield M, Yen L. Time to care? Health of informal older carers and time spent on health related activities: an Australian survey. BMC Public Health. 2013;13(1):374.
9. Butterworth P, Pymont C, Rodgers B, Windsor TD, Anstey KJ. Factors that Explain the Poorer Mental Health of Caregivers: Results from a Community Survey of Older Australians. Aust N Z J Psychiatry. 2010;44(7):616-24.
10. Hartmann ML, Wens J, Verhoeven V, Remmen R. The effect of caregiver support interventions for informal caregivers of community-dwelling frail elderly: systematic review. Int J Integr Care. 2012.
11. Litzelman K, Skinner H, Gangnon R, Nieto F, Malecki K, Witt W. The relationship among caregiving characteristics, caregiver strain, and health-related quality of life: evidence from the Survey of the Health of Wisconsin. Qual Life Res. 2015;24(6):1397-406.
12. Hughes N, Locock L, Ziebland S. Personal identity and the role of 'carer' among relatives and friends of people with multiple sclerosis. Soc Sci Med. 2013;96(Supp C):78-85.
13. Moore H, Gillespie A. The caregiving bind: Concealing the demands of informal care can undermine the caregiving identity. Soc Sci Med. 2014;116(Supp. C):102-9.
14. Charles L, Brémault-Phillips S, Parmar J, Johnson M, Sacrey L-A. Understanding how to support family caregivers of seniors with complex needs. Can Geriatr J. 2017;20(2):75-84.
15. Brown SHM, Abdelhafiz AH. Institutionalization of older people: Prediction and prevention. Aging Health. 2011;7(2):187-203.
16. Slatyer S, Toye C, Popescu A, Young J, Matthews A, Hill A, et al. Early re-presentation to hospital after discharge from an acute medical unit: Perspectives of older patients, their family caregivers and health professionals. J Clin Nurs. 2013;22(3-4):445-55.
17. AIHW. Admitted patient care 2015–16: Australian hospital statistics. 2017.
18. Bail K, Goss J, Draper B, Berry H, Karmel R, Gibson D. The cost of hospital-acquired complications for older people with and without dementia; a retrospective cohort study. BMC Health Serv Res. 2015;15(91).
19. Betini RSD, Hirdes JP, Lero DS, Cadell S, Poss J, Heckman G. A longitudinal study looking at and beyond care recipient health as a predictor of long term care home admission. BMC Health Serv Res. 2017;17(1):709.
20. AIHW. GEN aged care data factsheet: Government spending on aged care. 2017.
21. Toye C, Williamson DJ, Young J, Matthews A, Hill A, Gibson N. Reducing the early re-presentation of older adults to hospital after discharge from an Acute Assessment Unit: Determining predictors (Report). 2008.
22. Gonçalves-Bradley DC, Lannin NA, Clemson LM, Cameron ID, Shepperd S. Discharge planning from hospital. Cochrane Database Syst Rev. 2016(1).
23. Malouff J, Thorsteinsson E, Schutte N. The efficacy of problem solving therapy in reducing mental and physical health problems: A meta-analysis. Clin Psychol Rev. 2007;27:46-57.
24. Chi N-C, Demiris G. A systematic review of telehealth tools and interventions to support family caregivers. J Telemed Telecare. 2015;21(1):37-44.
25. Toye C, Parsons R, Slatyer S, Aoun SM, Moorin R, Osseiran-Moisson R, et al. Outcomes for family carers of a nurse-delivered hospital discharge intervention for older people (the Further Enabling Care at Home Program): Single blind randomised controlled trial. Int J Nurs Stud. 2016;64:32-41.
26. Hendrix CC, Bailey DE, Steinhauser KE, Olsen MK, Stechuchak KM, Lowman SG, . . . , et al. Effects of enhanced caregiver training program on cancer caregiver's self-efficacy, preparedness, and psychological well-being. Supportive Care in Cancer. 2016;24(1):327-36.
27. Duggleby W, Williams A, Ghosh S, Moquin H, Ploeg J, Markle-Reid M, et al. Factors influencing changes in health related quality of life of caregivers of persons with multiple chronic conditions. Health Qual Life Outcomes. 2016;14(1):81.
28. Youens D, Parsons R, Toye C, Slatyer S, Aoun S, Hill K, et al. Outcomes for older patients and the hospital system from implementing the Further Enabling Care at Home program for family caregivers: Findings from a randomised controlled trial Under review.

29. Richardson J, Khan MA, Iezzi A, Maxwell A. Comparing and Explaining Differences in the Magnitude, Content, and Sensitivity of Utilities Predicted by the EQ-5D, SF-6D, HUI 3, 15D, QWB, and AQoL-8D Multiattribute Utility Instruments. Med Decis Making. 2014;35(3):276-91.
30. Richardson J., Iezzi A., Khan M.A., A. M. Validity and reliability of the Assessment of Quality of Life (AQoL-8D) multi attribute utility instrument Patient. 2014;7(1):85-96.
31. Archbold PG, Stewart BJ, Greenlick MR, Harvath T. Mutuality and preparedness as predictors of caregiver role strain. Res Nurs Health. 1990;13(6):375-84.
32. Cooper B, Kinsella GJ, Picton C. Development and initial validation of a family appraisal of caregiving questionnaire for palliative care. Psychooncology. 2006;15(7):613-22.
33. Merluzzi T, Philip E, Vachon D, Heitzmann C. Assessment of self-efficacy for caregiving: The critical role of self-care in caregiver stress and burden. Palliat Support Care. 2011;9(1):15-24.
34. Aoun S, Monterosso L, Kristjanson L, McConigley R. Measuring symptom distress in palliative care: Psychometric properties of the Symptom Assessment Scale. J Palliat Med. 2011;14(3):315-21.
35. Yeo D, Faleiro R, Lincoln NB. Barthel ADL index: A comparison of administration methods. Clin Rehabil. 1995;9(1):34-9.
36. Western Australian Department of Health. Sustainable Health Review (Interim Report). 2018.
37. Aggar C, Ronaldson S, Cameron ID. Self-esteem in carers of frail older people: Resentment predicts anxiety and depression. Aging & Mental Health. 2011;15(6):671-8.
38. Maxwell A, Ozmen M, Iezzi A, Richardson J. Deriving population norms for the AQoL-6D and AQoL-8D multiattribute utility instruments from web-based data. Qual Life Res. 2016;25:3209-19.
39. Schumacher K, Stewart B, Archbold P, Caparro M, Mutale. F, Agrawal S. Effects of Caregiving Demand, Mutuality, and Preparedness on family caregiver outcomes during cancer treatment. Oncol Nurs Forum. 2008;35(1):49-56.
40. Henriksson A, Andershed B, Benzein E, Årestedt K. Adaptation and psychometric evaluation of the Preparedness for Caregiving Scale, Caregiver Competence Scale and Rewards of Caregiving Scale in a sample of Swedish family members of patients with life-threatening illness. Palliat Med. 2012;26(7):930-8.
41. Toye C, Walker H, Kristjanson L, Popescu A, Nightingale E. Measuring symptom distress among frail elders capable of providing self-reports. Nurs Health Sci. 2005;7(3):184-91.
42. Moore G, Audrey S, Barker M, Bond L, Bonell C, Hardeman W, et al. Process evaluation of complex interventions: Medical Research Council guidance BMJ. 2015;350.
43. Charlson M, Pompei P, Ales K, al. e. A new method of classifying prognostic comorbidity in longitudinal studies: Development and validation. J Chronic Dis. 1987;40:373–83.

PICF related documents:

For carers in WA when the patient provides their own consent:

WA site specific FECH Trial Carer PICF (including withdrawal form and summary)

DHS (now Services Australia) consent form

WA Carer Witnessed Verbal Consent Form (if unable to sign)

For patients in WA able to provide their own consent:

WA site specific FECH Trial Patient PICF (including withdrawal form and summary)

DHS (now Services Australia) consent form

WA Patient Witnessed Verbal Consent Form (if unable to sign)

For carers in WA when the patient is included via a waiver of consent:

WA site specific FECH Trial Carer PICF (including withdrawal form and summary)

DHS (now Services Australia) consent form

WA Carer Witnessed Verbal Consent Form (if unable to sign)

FECH Trial Opt Out Form and site specific WA Patient Information Sheet (including summary) - so that the patient may read the information sheet and summary and opt out if he or she regains capacity and wishes to do so.

For carers in Qld when the patient provides their own consent:

Qld FECH Trial Carer PICF (including withdrawal form and summary)

DHS (now Services Australia) consent form

Qld carer Witnessed Verbal Consent Form (if unable to sign)

For patients in Qld able to provide their own consent:

WA site specific FECH Trial Patient PICF (including withdrawal form and summary)

DHS (now Services Australia) consent form

WA Patient Witnessed Verbal Consent Form (if unable to sign)

For carers in Qld when the patient is included via consent from a substitute decision maker:

Qld FECH Trial Carer PICF (including withdrawal form and summary)

DHS (now Services Australia) consent form

Qld Carer Witnessed Verbal Consent Form (if unable to sign)

Site specific substitute decision maker for patient (SDM) PICF (includes form for SDM to withdraw the patient and summary)

DHS (now Services Australia) consent form for the patient if the carer has the authority to sign this on the patient's behalf (as specified on the form)

FECH Trial Opt Out Form and Qld Patient Information Sheet (includes summary) so that the patient may read the information sheet and opt out if he or she regains capacity and wishes to do so.

For FECH Nurses:

FECH Nurse PICF (including withdrawal form)
