# Supplementary material for: Nurse Telephone Support for Caregivers of Older Adults at Hospital Discharge: A Randomized Clinical Trial
Source: JAMA Netw Open. 2024 Oct 25;7(10):e2441019. doi: 10.1001/jamanetworkopen.2024.41019 (PMC11581515; doi:10.1001/jamanetworkopen.2024.41019)
Supplement: Supplement 2. — eTable 1. TIDieR Checklist for FECH+ Intervention eFigure 1. Structure of the AQoL-8D eMethods. Secondary Outcome Measures eFigure 2. Changes in Health-Related Quality of Life AQoL-8D: Overall Utility Score eFigure 3. Changes in Health-Related Quality of Life AQoL-8D: Psychosocial Super Dimension eFigure 4. Changes in Health-Related Quality of Life AQoL-8D: Psychosocial Subscales a) Mental Health; b) Happiness; c) Self-Worth eFigure 5. Changes in Health-Related Quality of Life AQoL-8D: Psychosocial Subscales d) Coping; e) Relationships eTable 2. Changes in Health-Related Quality of Life AQoL-8D: Psychosocial Subscales a) Mental Health; b) Happiness; c) Self-Worth; d) Coping; e) Relationships eFigure 6. Changes in Health-Related Quality of Life AQoL-8D: Physical Super Dimension eTable 3. Changes in Health-Related Quality of Life AQoL-8D: Physical Super Dimension eFigure 7. Changes in Health-Related Quality of Life AQoL-8D: Physical Subscales a) Independent Living; b) Senses; c) Pain eTable 4. Changes in Health-Related Quality of Life AQoL-8D: Physical Subscales a) Independent Living; b) Senses; c) Pain eTable 5. AQoL-8D: Summary Statistics eTable 6. Secondary Outcomes Summary Statistics: Preparedness for Care (PCS), Caregiver Inventory (CGI), Family Appraisal of Caregiving Questionnaire (FACQ) [file jamanetwopen-e2441019-s002.pdf]

Supplemental Online Content

Hill A-M, Moyle W, Slatyer S, et al. Nurse telephone support for caregivers of older adults at hospital discharge: a randomized clinical trial. *JAMA Netw Open*. 2024;7(10):e2441019. doi:10.1001/jamanetworkopen.2024.41019

eTable 1. TIDieR Checklist for FECH+ Intervention

eReferences

eFigure 1. Structure of the AQoL-8D

eMethods. Secondary Outcome Measures

eFigure 2. Changes in Health-Related Quality of Life AQoL-8D: Overall Utility Score

eFigure 3. Changes in Health-Related Quality of Life AQoL-8D: Psychosocial Super Dimension

eFigure 4. Changes in Health-Related Quality of Life AQoL-8D: Psychosocial Subscales a) Mental Health; b) Happiness; c) Self-Worth

eFigure 5. Changes in Health-Related Quality of Life AQoL-8D: Psychosocial Subscales d) Coping; e) Relationships

eTable 2. Changes in Health-Related Quality of Life AQoL-8D: Psychosocial Subscales a) Mental Health; b) Happiness; c) Self-Worth; d) Coping; e) Relationships

eFigure 6. Changes in Health-Related Quality of Life AQoL-8D: Physical Super Dimension

eTable 3. Changes in Health-Related Quality of Life AQoL-8D: Physical Super Dimension

eFigure 7. Changes in Health-Related Quality of Life AQoL-8D: Physical Subscales a) Independent Living; b) Senses; c) Pain

eTable 4. Changes in Health-Related Quality of Life AQoL-8D: Physical Subscales a) Independent Living; b) Senses; c) Pain

eTable 5. AQoL-8D: Summary Statistics

eTable 6. Secondary Outcomes Summary Statistics: Preparedness for Care (PCS), Caregiver Inventory (CGI), Family Appraisal of Caregiving Questionnaire (FACQ)

This supplemental material has been provided by the authors to give readers additional

information about their work.

© 2024 Hill A-M et al. *JAMA Network Open*

eTable 1. TIDieR Checklist[1] for FECH+ Intervention

**1. Brief name**	Further Enabling Care at Home (FECH+) for informal caregivers of older adults discharged home from hospital.
**2. Why**	The FECH+ program offered a problem-solving, caregiver-focussed approach to improve outcomes for the caregiver and care recipient and was additional to usual care. It was designed to provide caregivers with timely health professional support and training to use the resources available in the community. It aimed to develop problem-solving skills and address the caregiver's identified needs. Resources provided to individual caregivers as required, included information and contact details for Carers' national and local support organisations, My Aged Care (national government agency providing aged care services), Aged care medical assessment teams, Home care services, and Dementia community support groups.
**3. What - materials**	All caregivers (100%) completed the Carer Support Needs Assessment Tool (CSNAT) with support provided by the FECH+ nurse. A standard operating procedures manual was used by the nurse. All contact between the participant and the nurse was by telephone call. **Call 1:** the FECH+ nurse first elicited the caregiver's understanding of information provided to them, at the time of the discharge, by the hospital. If the caregiver demonstrated insufficient understanding of discharge information to form the basis for caregiving, the FECH+ nurse guided the caregiver to access the information required. The CSNAT was provided to the caregiver, via email / mail, to allow the caregiver time to reflect upon the included items before Call 2. The FECH+ Program Caregiver Booklet, which summarized key elements of the program, was also provided between Calls 1 and 2. **Call 2**: the FECH+ nurse guided the caregiver through the reflective CSNAT approach, using the CSNAT to identify and prioritize their support needs. **Call 3-6**: the caregiver was prompted to reflect upon the extent to which support was accessed as planned since the previous call, then again use the CSNAT approach combined with problem-solving to identify, prioritize, and address remaining or additional support needs. Resources relevant for individual caregivers were subsequently emailed / mailed to participants.
**4. What - procedures**	The FECH+ nurse facilitated caregivers to (a) reflect upon the current caregiving situation, (b) identify and prioritize new or ongoing support needs, and (c) implement a problem-solving approach to address these support needs. Caregivers were guided to address up to three prioritized needs using problem-solving techniques and goal setting.<br>The first call was designed to introduce the nurse and the phone call plan for the next 6 months together with an explanation of the ADAPT approach to problem solving.[2,3] The theoretical basis for the intervention was Problem Solving Therapy[2] using the ADAPT approach,[3] which emphasized the role of the caregiver as the problem solver.<br>During subsequent phone calls caring responsibilities were discussed, with 100% of participants using the CSNAT to identify problems. The program aimed to facilitate the development of caregivers' problem-solving skills to continue without support from the FECH+ nurse after the intervention was completed. Each call provided an opportunity to reinforce the problem-solving skills learnt.
**5. Who provided**	Registered nurses experienced in gerontological nursing and who received training in delivering the FECH+ program.
**6. How**	Delivered via telephone to the caregiver after the care recipient was discharged from hospital.
**7. Where**	Delivered directly to the caregiver via their phone.
**8. When and how much**	Six telephone calls by the FECH+ nurse after the care recipient's discharge from hospital. Call plan: *Call 1*) during the first week after discharge (15 minutes); *Call 2*) at two weeks after discharge (approximately 45 minutes);

© 2024 Hill A-M et al. *JAMA Network Open*

	Calls 3 to 6) at 1, 2, 4, and 6 months respectively after discharge, (each approximately 30 minutes). The introduction call median (IQR) duration was 12 (8-17) minutes. Most time was spent on the second call when problem solving discussion commenced [median call duration = 33 (23-50) minutes]. The least time was spent on the 6th (final) call [median call duration = 16 (10-30) minutes].
**9. Tailoring**	The intervention was tailored to the needs of each caregiver, using a problem-solving approach to identify, prioritize, and address the top three support needs. Individual support was provided to participants according to problems or needs identified.
**10. Modifications**	The intervention was delivered as intended with no modifications.
**11. Fidelity**	All participants received some component of the intervention. Six telephone calls were made to 222 (81.0%) intervention group participants and 95.3% of participants received at least 4 calls. Resources (such as information about receiving caregiver support) were issued to 243 (88.7%) participants on at least one phone call.
**12. Adherence**	Of the 274 participants, 267 (97.4%) indicated priority concerns to be discussed during phone calls. The 7 participants who did not list any priorities, indicated they were managing well and required no extra support. The two most common priorities discussed were practical help in the home and managing relative's symptoms, including giving medication. (*Note: Participants' response to the intervention to be reported in detail in a further process evaluation*).
Notes: CSNAT = Carer Support Needs Assessment Tool	

eReferences

1. Hoffmann T, Glasziou P, Boutron I, et al. Better reporting of interventions: template for intervention description and replication (TIDieR) checklist and guide. BMJ. 2014;348:g1687
2. Beaudreau SA, Gould CE, Sakai E, Huh JWT. Problem-solving therapy. Encyclopedia of Geropsychology. 2015; doi: 10.1007/978-981-287-080-3_90-1
3. Nezu AM, Nezu CM, D'Zurilla TJ. Solving Life's Problems: a 5-Step Guide to Enhanced Well-Being. (1st ed.). New York, NY: Springer Publishing Company; 2007.

© 2024 Hill A-M et al. *JAMA Network Open*

eFigure 1. Structure of the AQoL-8D

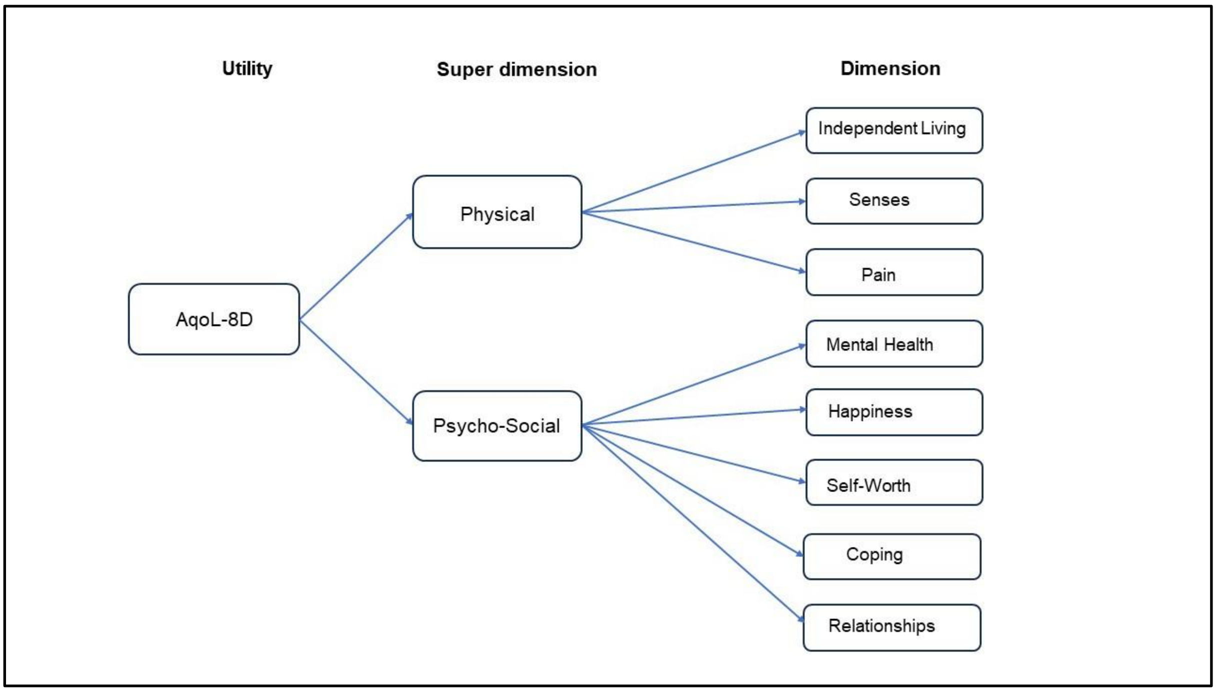

© 2024 Hill A-M et al. *JAMA Network Open*

eMethods. Secondary Outcome Measures

1. Preparedness to Care

Measured using the *Preparedness for Caregiving Scale (PCS)* - an 8-item questionnaire used to determine caregivers' perceptions about their readiness to provide physical, emotional and instrumental care, along with managing the stress of caregiving for the care recipient.[1] Each of the 8 items in the PCS has 5 response options (ranging from 0 = not at all prepared, to 4 = very well prepared). The PCS is scored by calculating the mean of all items answered, therefore higher scores indicate greater preparedness for caregiving (*mean score ranges from 0 to 4*).

The PCS has been evaluated for use in caregivers of older adults including African American caregivers, caregivers in Korea and in populations of care recipients including stroke, heart failure, coronary artery disease, dementia and cancer.[1-7] The construct validity for the PCS has been established in older adults.[8] Testing in patient populations with life-threatening illness, dementia and stroke has confirmed satisfactory internal consistency, test-retest reliability and stability and unidimensionality of the PCS.[5,9,10]

2. Caregiver Self-Efficacy

Measured using the *Caregiver Inventory (CGI)* - a 21-item questionnaire used to assess caregiving self-efficacy expectations.[11] Self-efficacy is built through mastery of tasks and ability to persist. The CGI has four subscales: i) managing medical information, ii) caring for the care recipient, iii) caring for oneself and iv) managing difficult interactions and emotions. Each of the 21 items is scored using a 9-point Likert scale (range; 1 = not at all confident to 9 = totally confident), therefore higher scores indicate better self-efficacy. The CGI is scored by summing the score of each item and the *total score range is from 21 to 189.*

The CGI has been used in studies conducted in Italian, United States, Chinese and Indonesian populations.[11-16] Evaluation of the CGI has established the tool as a reliable and validated measure of caregiver self-efficacy in United States, Italian and Chinese populations.[11-13] No ceiling or floor effects were observed in the Chinese evaluation of the CGI.[13] The four CGI subscales have been confirmed by factor analysis.[12] Cronbach's alpha for the CGI was 0.91 in a sample of caregivers of patients for whom the main diagnoses were cancer, chronic obstructive pulmonary disease, stroke, chronic heart failure and dementia.[12]

3. Caregiver Strain and Distress

Measured with the *Family Appraisal of Caregiving Questionnaire (FACQ)*, which was developed for caregivers of adults receiving palliative care.[17] The FACQ has four subscales: i) caregiver strain, ii) positive caregiving appraisals, iii) caregiver distress and iv) family well-being. We used two sub-scales of the FACQ (sub-scales one and three) that have eight and five items respectively.[17] Each item is scored using a 5-point Likert scale, (range; 1 = strongly agree to 5 = strongly disagree). Each sub-scale is totalled separately and the score recorded and *the total range is from 5 to 25 and 8 to 30 for each sub scale,* therefore, higher scores indicate less levels of strain and distress.

The FACQ has been used in randomised trials evaluating experiences of caregivers of adults with stroke, palliative care, chronic obstructive pulmonary disease in Denmark, the United Kingdom, and The Netherlands.[18-20] The FACQ has been established in psychometric studies as a valid and reliable measure of caregiver experience in Australian and Chinese caregivers of adults with cancer.[17,21]

eReferences

1. Archbold PG, Stewart BJ, Greenlick MR, Harvath T. Mutuality and preparedness as predictors of caregiver role strain. *Res Nurs Health.* 1990;13(6):375–384. doi: 10.1002/nur.4770130605.
2. Petruzzo A, Paturzo M, Buck HG, et al. Psychometric evaluation of the caregiver preparedness scale in caregivers of adults with heart failure. *Res Nurs Health*. 2017;40:470–8. doi: 10.1002/nur.21811
3. Grant M, Sun V, Fujinami R, et al. Family caregiver burden, skills preparedness, and quality of life in non-small cell lung cancer. *Oncol Nurs Forum*. 2013;40:337–46. doi: 10.1188/13.ONF.337-346 21.
4. Kneeshaw MF, Considine RM, Jennings J. Mutuality and preparedness of family caregivers for elderly women after bypass surgery. *Appl Nurs Res.* 1999;12:128–35. doi: 10.1016/s0897-1897(99)80034-2
5. Pucciarelli G, Savini S, Byun E, et al. Psychometric properties of the caregiver preparedness scale in caregivers of stroke survivors. *Heart Lung*. 2014;43(6):555–560. doi: 10.1016/j.hrtlng.2014.08.004

© 2024 Hill A-M et al. *JAMA Network Open*

6. Kuzmik A, Boltz M, Resnick B, BeLue R. Evaluation of the caregiver preparedness scale in African American and white caregivers of persons with dementia during post-hospitalization transition. *J Nurs Meas*. 2021;JNM-D-20-00087. advance online publication. doi: 10.1891/JNM-D-20-00087
7. Uhm KE, Jung H, Woo MW, et al. Influence of preparedness on caregiver burden, depression, and quality of life in caregivers of people with disabilities. *Front Public Health*. 2023;11:1153588. doi: 10.3389/fpubh.2023.1153588
8. Schumacher KL, Stewar BJ, Archbold PG, Caparro M, Mutale F, Agrawal S. Effects of caregiving demand, mutuality, and preparedness on family caregiver outcomes during cancer treatment. *Oncol Nurs Forum*. 2008;35(1):49–56. doi:10.1188/08.ONF.49-56
9. Henriksson A, Andershed B, Benzein E, Arestedt K. Adaptation and psychometric evaluation of the preparedness for caregiving scale, caregiver competence scale and rewards of caregiving scale in a sample of Swedish family members of patients with life-threatening illness. *Palliat Med*. 2012;26(7):930–938. doi: 10.1177/0269216311419987
10. Gutierrez-Baena B, Romero-Grimaldi C. Predictive model for the preparedness level of the family caregiver. *Int J Nurs Pract*. 2022;28:e13057. doi: 10.1111/ijn.13057
11. Merluzzi T, Philip E, Vachon D, Heitzmann C. Assessment of self-efficacy for caregiving: the critical role of self-care in caregiver stress and burden. *Palliat Support Care*. 2011;9(1):15-24. doi: 10.1017/S1478951510000507
12. Serpentini S, Guandalini B, Tosin G, et al. Assessment of self-efficacy for caregiving in oncology: Italian validation of the caregiver inventory (CGI-I). *BMC Palliat Care.* 2021;20(1):166. doi:10.1186/s12904-021-00849-5
13. Leung DYP, Chan HYL, Chan CWH, et al. Psychometric properties of the caregiver inventory for measuring caregiving self-efficacy of caregivers of patients with palliative care needs. *Neuropsychiatry (London).* 2017;7(6):872–879.
14. Mazanec SR, Sattar A, Delaney CP, Daly BJ. Activation for health management in colorectal cancer survivors and their family caregivers. *West J Nurs Res.* 2016;38(3):325-344. doi:10.1177/0193945915604055
15. Semere W, Althouse AD, Arnold R, et al. Examining caregiver outcomes in the CONNECT intervention for patients with advanced cancer. *J Pain Symptom Manage*. 2023;65(3):173-182. doi: 10.1016/j.jpainsymman.2022.11.025.
16. Rochmawati E, Saun AU. Effectiveness of symptom management training on caregiving preparedness and burden of family caregivers in-home palliative care: a quasi- experimental study. *Jurnal ners*. 2022;17(2):153-160. doi: 10.20473/jn.v17i2.38147
17. Cooper B, Kinsella GJ, Picton C. Development and initial validation of a family appraisal of caregiving questionnaire for palliative care. *Psychooncology.* 2006;15(7):613-22. doi:10.1002/pon.1001
18. Patchwood E, Woodward-Nutt K, Rhodes SA, et al. Organising Support for Carers of Stroke Survivors (OSCARSS): a cluster randomised controlled trial with economic evaluation. *BMJ Open.* 2021;11(1):e038777. doi:10.1136/bmjopen-2020-038777 31
19. Lund L, Ross L, Petersen MA, et al. (2020). Effect of the Carer Support Needs Assessment Tool intervention (CSNAT-I) in the Danish specialised palliative care setting: a stepped-wedge cluster randomised controlled trial. *BMJ Support Palliat Care.* 2020;bmjspcare-2020-002467. Advance online publication. doi: 10.1136/bmjspcare-2020-002467
20. Nakken N, Janssen DJ, van Vliet M, et al. Gender differences in partners of patients with COPD and their perceptions about the patients. *Int J Chron Obstruct Pulmon Dis*. 2017;12:95–104. doi: 10.2147/COPD.S118871
21. Zhang S, Zhang S, Qin Y, Wan Q. Reliability and validity of the Chinese version of the family appraisal of caregiving questionnaire for palliative care. *Chinese Nursing Management*. 2022;22(6):863-868. doi: 10.3969/j.issn.1672-1756.2022.06.013

© 2024 Hill A-M et al. *JAMA Network Open*

eFigure 2. Changes in Health-Related Quality of Life AQoL-8D: Overall Utility Score

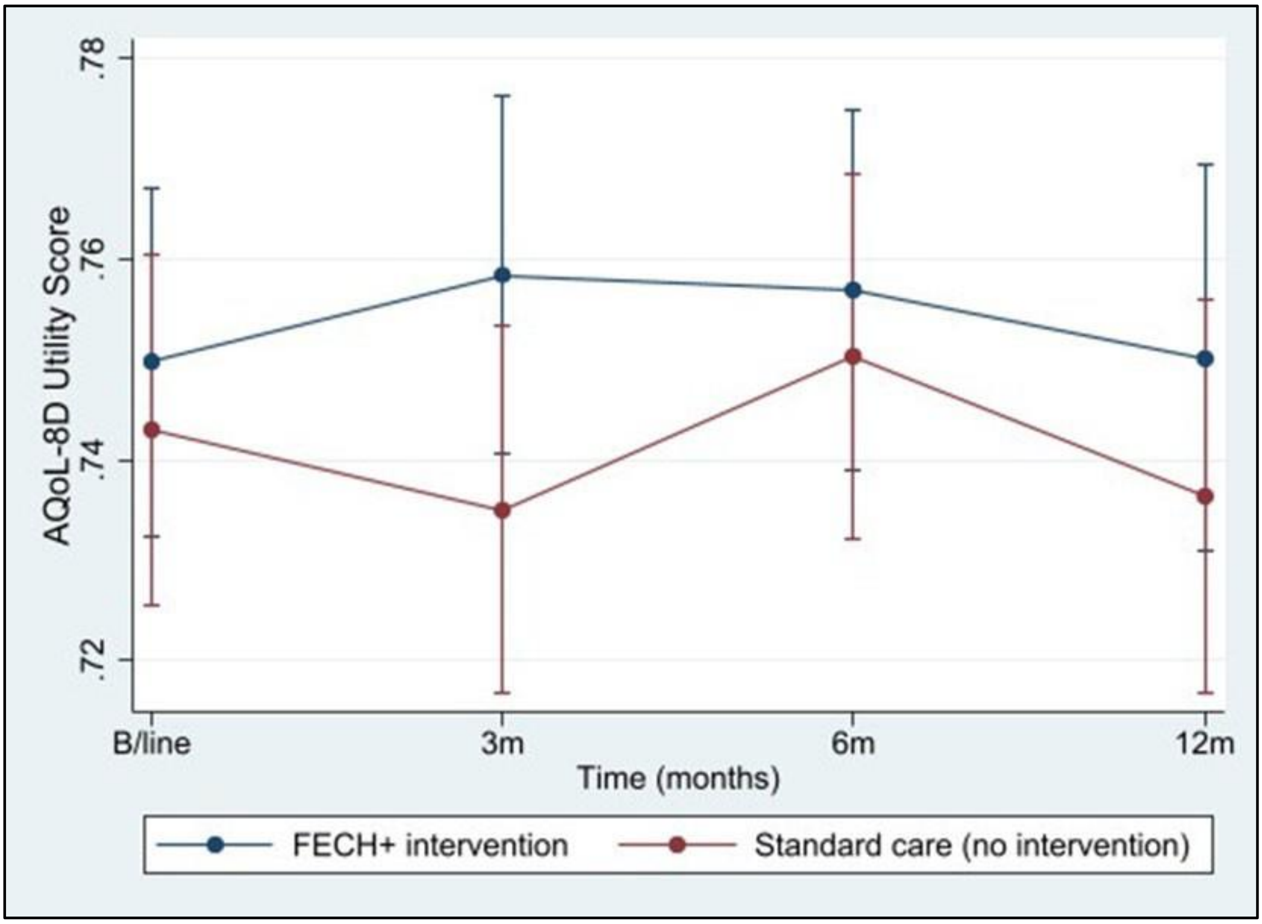

© 2024 Hill A-M et al. *JAMA Network Open*

eFigure 3. Changes in Health-Related Quality of Life AQoL-8D: Psychosocial Super Dimension

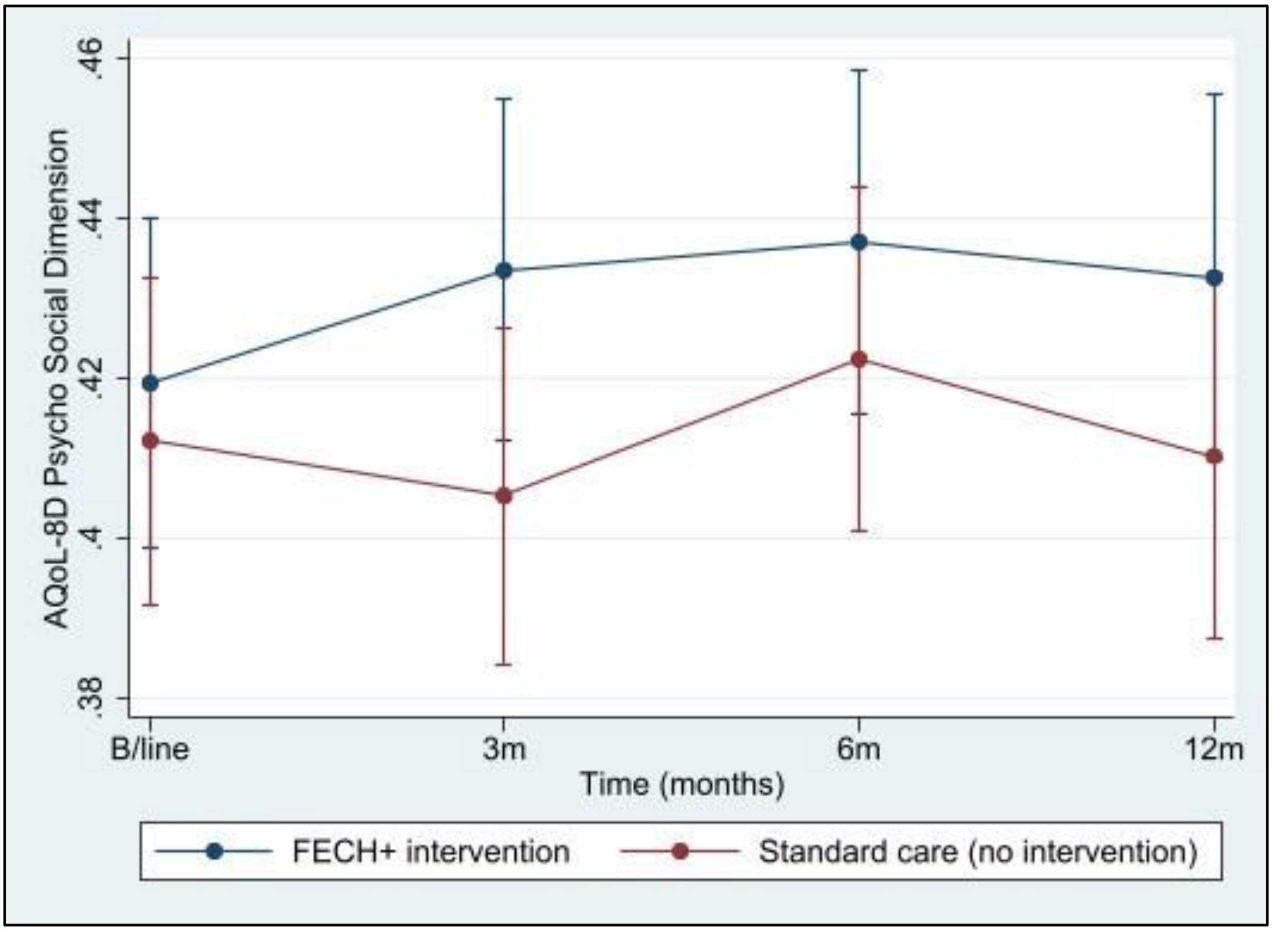

eFigure 4. Changes in Health-Related Quality of Life AQoL-8D: Psychosocial Subscales a) Mental Health; b) Happiness; c) Self-Worth

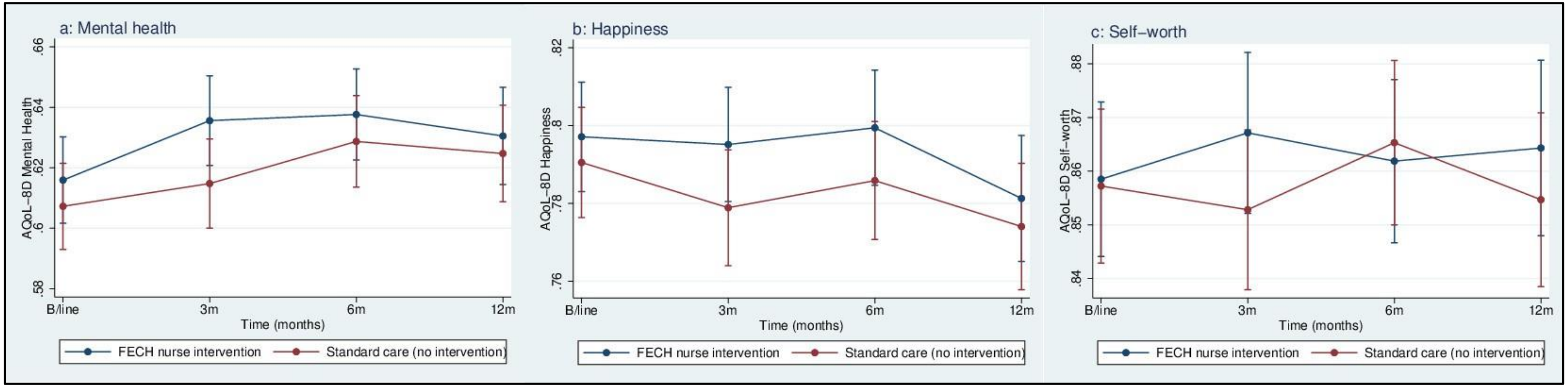

© 2024 Hill A-M et al. *JAMA Network Open*

eFigure 5. Changes in Health-Related Quality of Life AQoL-8D: Psychosocial Subscales d) Coping; e) Relationships

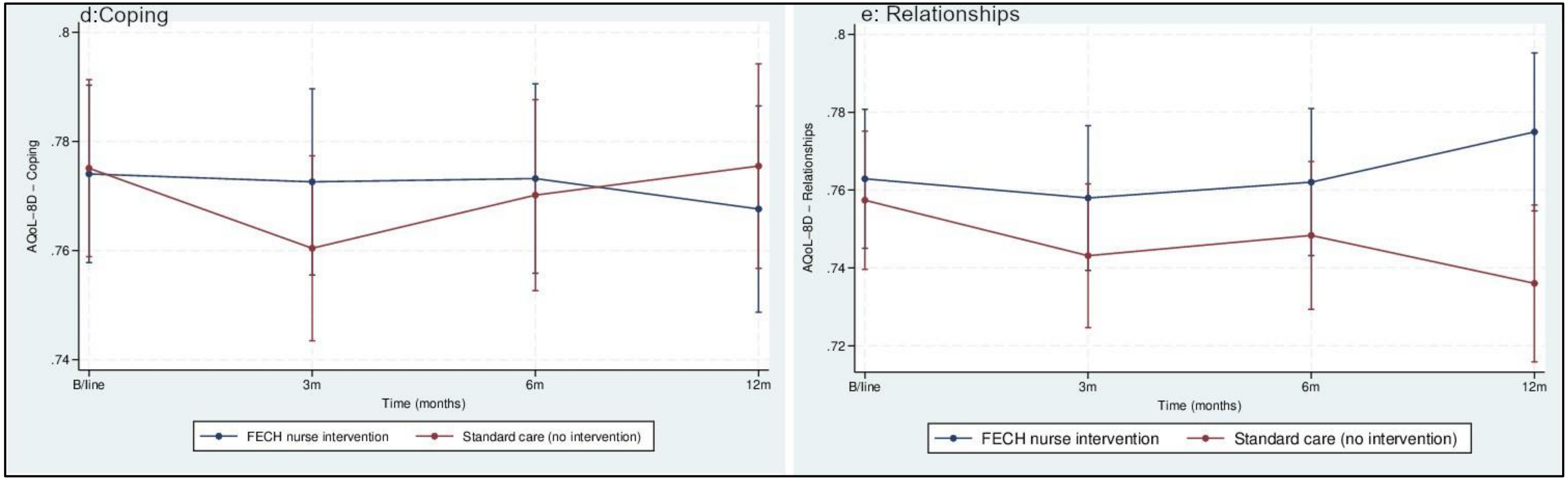

© 2024 Hill A-M et al. *JAMA Network Open*

eTable 2. Changes in Health-Related Quality of Life AQoL-8D: Psychosocial Subscales a) Mental Health; b) Happiness; c) Self-Worth; d) Coping; e) Relationships

**Sub scale**	**Time**	**Intervention** mean (95% CI)	**Change from baseline (within group)** mean (95% CI)	**p#**	**Control** mean (95% CI)	**Change from baseline (within group)** mean (95% CI)	**p#**	**Difference between groups** mean (95% CI)	**p**	**p***
**Mental health**	B	0.62 (0.60,0.63)			0.61 (0.59,0.62)			-0.009 (-0.029, 0.012)	0.401	
	3m	0.64 (0.62,0.65)	0.020 (0.007,0.032)	0.002	0.61 (0.60,0.63)	0.008 (-0.005, 0.020)	0.240	-0.021 (-0.042, 0.000)	0.052	0.181
	6m	0.64 (0.62,0.65)	0.022 (0.009,0.035)	0.001	0.63 (0.61,0.64)	0.021 (0.008, 0.034)	0.001	-0.009 (-0.030, 0.013)	0.415	0.979
	12m	0.63 (0.61,0.65)	0.015 (0.001,0.029)	0.042	0.63 (0.61,0.64)	0.017 (0.003, 0.031)	0.014	-0.006 (-0.029, 0.017)	0.617	0.778
**Happiness**	B	0.80 (0.78,0.81)			0.79 (0.78,0.80)			-0.007 (-0.027, 0.013)	0.518	
	3m	0.80 (0.78,0.80)	-0.003 (-0.023,0.017)	0.754	0.78 (0.76,0.79)	-0.018 (-0.038, 0.001)	0.069	-0.016 (-0.037, 0.005)	0.130	0.288
	6m	0.80 (0.78,0.81)	0.004 (-0.016,0.024)	0.718	0.79 (0.77,0.8)	-0.007 (-0.028, 0.013)	0.480	-0.014 (-0.035, 0.008)	0.211	0.449
	12m	0.78 (0.77,0.80)	-0.025(-0.047,-0.003)	0.026	0.77 (0.76,0.79)	-0.026 (-0.047, -0.004)	0.021	-0.007 (-0.030, 0.016)	0.538	0.962
**Self-worth**	B	0.86 (0.84, 0.87)			0.86 (0.84, 0.87)			-0.001 (-0.022, 0.019)	0.902	
	3m	0.87 (0.85, 0.88)	0.009 (-0.004, 0.022)	0.196	0.85 (0.84,0.87)	-0.004 (-0.017, 0.009)	0.506	-0.014 (-0.036, 0.007)	0.186	0.166
	6m	0.86 (0.85, 0.88)	0.003 (-0.010, 0.017)	0.621	0.87 (0.85,0.88)	0.008 (-0.005, 0.022)	0.241	0.003 (-0.018, 0.025)	0.756	0.626
	12m	0.86 (0.85, 0.88)	0.006 (-0.009, 0.020)	0.436	0.85 (0.84, 0.87)	-0.003 (-0.017, 0.012)	0.731	-0.010 (-0.033, 0.013)	0.414	0.426
**Coping**	B	0.77 (0.76,0.79)			0.78 (0.76,0.79)			0.001 (-0.022, 0.024)	0.929	
	3m	0.77 (0.76,0.79)	-0.001 (-0.018,0.015)	0.864	0.76 (0.74,0.78)	-0.015 (-0.031, 0.002)	0.080	-0.012 (-0.036, 0.012)	0.323	0.265
	6m	0.77 (0.76,0.79)	-0.001 (-0.018,0.016)	0.921	0.77 (0.75,0.79)	-0.005 (-0.022,0.012)	0.570	-0.003 (-0.028,0.022)	0.810	0.738

© 2024 Hill A-M et al. *JAMA Network Open*

**Sub scale**	**Time**	**Intervention** mean (95% CI)	**Change from baseline (within group)** mean (95% CI)	**p#**	**Control** mean (95% CI)	**Change from baseline (within group)** mean (95% CI)	**p#**	**Difference between groups** mean (95% CI)	**p**	**p***
	12m	0.77 (0.75,0.79)	-0.006 (-0.025,0.012)	0.493	0.78 (0.76,0.79)	0.000 (-0.018,0.019)	0.965	0.008 (-0.019, 0.035)	0.563	0.605
**Relation ships**	B	0.76 (0.75,0.78)			0.76 (0.74,0.78)			-0.006 (-0.031, 0.020)	0.669	
	3m	0.76 (0.74,0.78)	-0.005 (-0.021,0.012)	0.559	0.74 (0.72,0.76)	-0.014 (-0.031,0.002)	0.089	-0.015 (-0.041, 0.012)	0.270	0.433
	6m	0.76 (0.74,0.78)	-0.001 (-0.018,0.016)	0.920	0.75 (0.73,0.77)	-0.009 (-0.026,0.008)	0.298	-0.014 (-0.041,0.013)	0.319	0.503
	12m	0.77 (0.75,0.80)	0.012 (-0.006,0.030)	0.201	0.74 (0.74,0.76)	-0.021 (-0.040,-0.003)	0.022	-0.039 (-0.068,-0.010)	0.008	0.012

Note: B=baseline; m=months; CI=confidence interval; p# = within group change from baseline; p = cross-sectional mean difference; p* = interaction effect (difference of the differences between groups)

© 2024 Hill A-M et al. *JAMA Network Open*

eFigure 6. Changes in Health-Related Quality of Life AQoL-8D: Physical Super Dimension

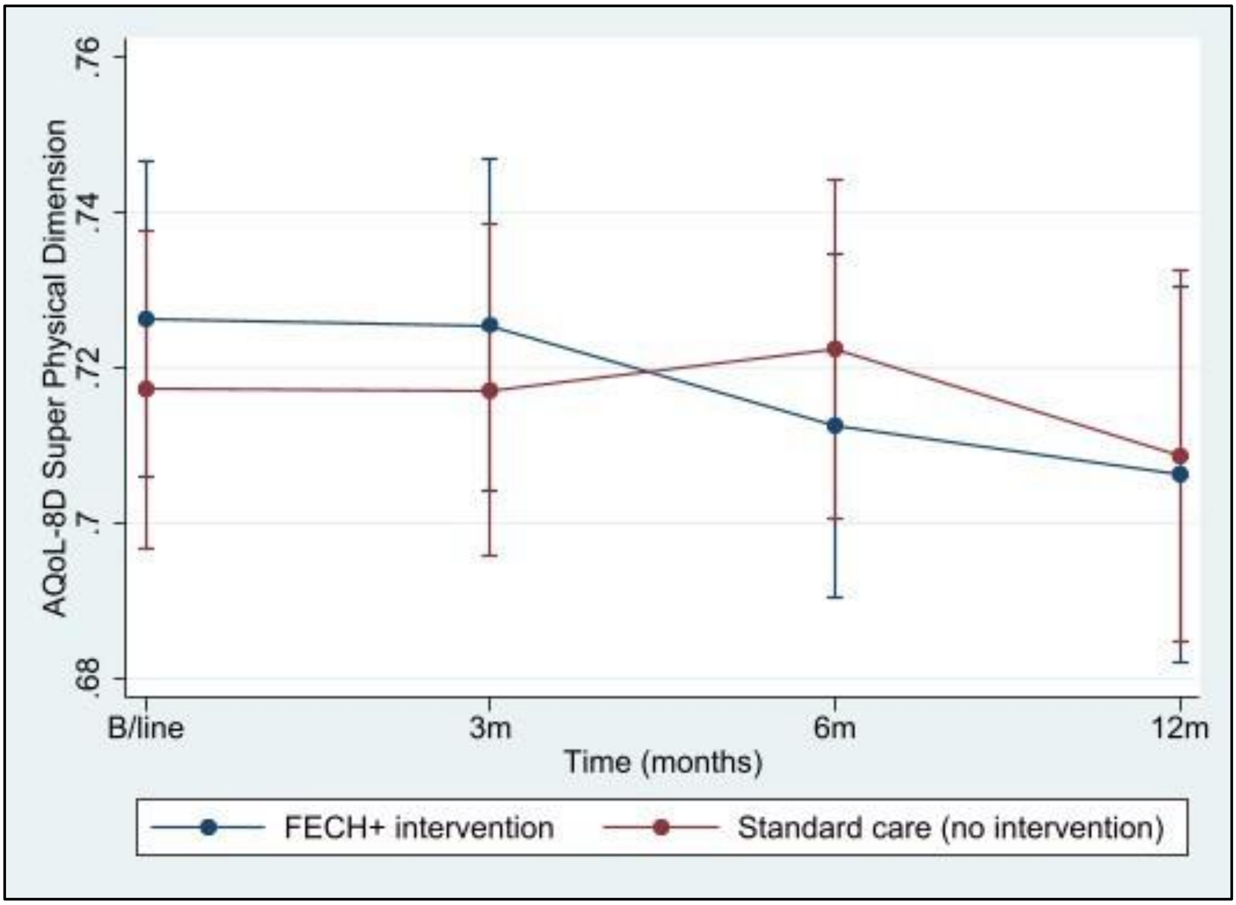

eTable 3. Changes in Health-Related Quality of Life AQoL-8D: Physical Super Dimension

**Time**	**Intervention** mean (95% CI)	**Change from baseline (within group)** mean (95% CI)	**p#**	**Control** mean (95% CI)	**Change from baseline (within group)** mean (95% CI)	**p#**	**Difference between groups** mean (95% CI)	**p**	**p***
B	0.73 (0.71,0.75)			0.72 (0.70,0.74)			-0.009 (-0.038,0.020)	0.536	
3m	0.73 (0.70,0.75)	-0.001 (-0.029,0.027)	0.940	0.72 (0.70,0.74)	0.000 (-0.028,0.028)	0.992	-0.008 (-0.039,0.022)	0.584	0.963
6m	0.71 (0.69,0.73)	-0.019 (-0.048,0.009)	0.189	0.72 (0.70,0.74)	0.007 (-0.022,0.036)	0.615	0.010 (-0.021,0.041)	0.531	0.200
12m	0.71 (0.68,0.73)	-0.028 (-0.059,0.004)	0.082	0.71 (0.68,0.73)	-0.012 (-0.043,0.019)	0.453	0.002 (-0.032,0.036)	0.894	0.480

Note: B=baseline; m=months; CI=confidence interval; p# = within group change from baseline; p = cross-sectional mean difference; p* = interaction effect (difference of the differences between groups)

© 2024 Hill A-M et al. *JAMA Network Open*

eFigure 7. Changes in Health-Related Quality of Life AQoL-8D: Physical Subscales a) Independent Living; b) Senses; c) Pain

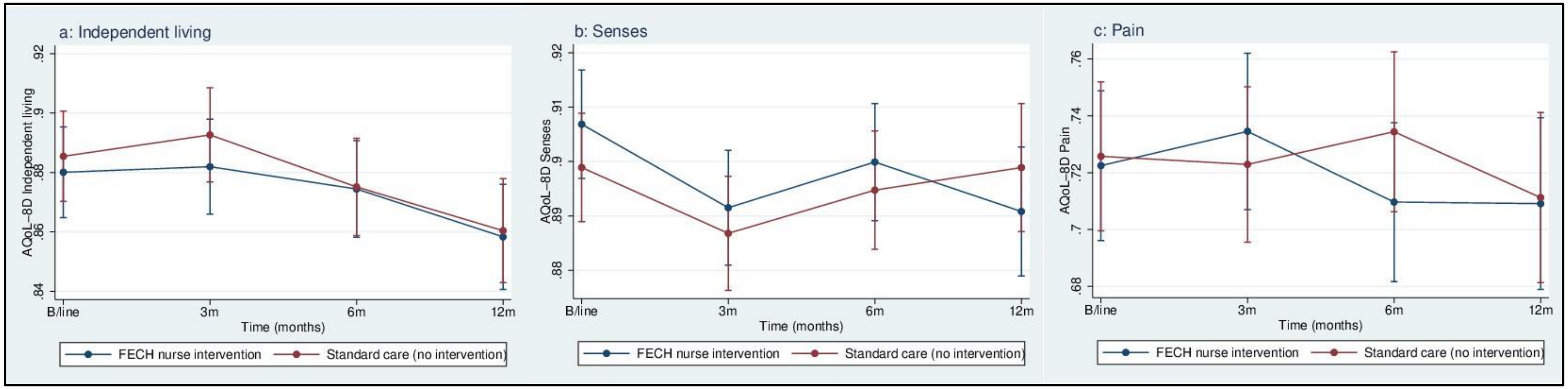

© 2024 Hill A-M et al. *JAMA Network Open*

eTable 4. Changes in Health-Related Quality of Life AQoL-8D: Physical Subscales a) Independent Living; b) Senses; c) Pain

Subscale	Time	**Intervention** mean (95% CI)	**Change from baseline (within group)** mean (95% CI)	p#	**Control** mean (95% CI)	**Change from baseline (within group)** mean (95% CI)	p#	**Difference between groups** mean (95% CI)	p	p*
**Independent living**	B	0.88 (0.86,0.90)			0.89 (0.87, 0.90)			0.005 (-0.016, 0.027)	0.626	
	3m	0.88 (0.87, 0.90)	0.002 (-0.013, 0.017)	0.810	0.89 (0.88, 0.91)	0.007 (-0.008, 0.022)	0.355	0.011 (-0.012, 0.033)	0.354	0.629
	6m	0.87 (0.86, 0.89)	-0.006 (-0.021, 0.010)	0.480	0.88 (0.86, 0.89)	-0.010 (-0.026, 0.005)	0.201	0.001 (-0.022, 0.024)	0.953	0.680
	12m	0.86 (0.84, 0.88)	-0.022 (-0.039, -0.005)	0.013	0.86 (0.84, 0.88)	-0.025 (-0.042, -0.008)	0.004	0.002 (-0.023, 0.027)	0.871	0.788
**Senses**	B	0.91 (0.90,0.92)			0.90 (0.89,0.91)			-0.008 (-0.022, 0.006)	0.270	
	3m	0.89 (0.88,0.90)	-0.015 (-0.026, -0.004)	0.007	0.89 (0.88,0.90)	-0.012 (-0.023, -0.001)	0.033	-0.005 (-0.020, 0.010)	0.537	0.687
	6m	0.90 (0.89,0.91)	-0.007 (-0.018, 0.004)	0.231	0.89 (0.88,0.91)	-0.004 (-0.016, 0.007)	0.478	-0.005 (-0.021, 0.010)	0.510	0.735
	12m	0.89 (0.88,0.90)	0.016 (-0.028, -0.004)	0.011	0.90 (0.89,0.91)	0.000 (-0.012, 0.012)	0.998	0.008 (-0.009, 0.025)	0.345	0.073
**Pain**	B	0.72 (0.70, 0.75)			0.73 (0.70, 0.75)			0.003 (-0.034, 0.041	0.863	
	3m	0.73 (0.71, 0.76)	0.012 (-0.013, 0.037)	0.346	0.72 (0.70, 0.75)	-0.003 (-0.028, 0.022)	0.822	-0.012 (-0.051, 0.027)	0.559	0.409
	6m	0.71 (0.68, 0.74)	-0.013 (-0.038, 0.013)	0.326	0.73 (0.71, 0.76)	0.009 (-0.017, 0.034)	0.512	0.025 (-0.015, 0.065)	0.223	0.247
	12m	0.71 (0.68, 0.74)	-0.013 (-0.041, 0.015)	0.349	0.71 (0.68, 0.74)	-0.015 (-0.042, 0.013)	0.305	0.002 (-0.041, 0.045)	0.922	0.954

Note: B=baseline; m=months; CI=confidence interval; p# = within group change from baseline; p = cross-sectional mean difference; p* = interaction effect (difference of the differences between groups)

© 2024 Hill A-M et al. *JAMA Network Open*

eTable 5. AQoL-8D: Summary Statistics

	FECH+ Intervention; n=274				Control; n=273			
**Time Point**	**Baseline (n=274)**	**3 Months (n=262)**	**6 Months (n=266)**	**12 Months (n=246)**	**Baseline (n=273)**	**3 Months (n=258)**	**6 Months (n=261)**	**12 Months (n=235)**
**AQoL Utility**	0.73 (0.17)*	0.74 (0.18)	0.74 (0.18)	0.73 (0.19)	0.73 (0.17)	0.72 (0.18)	0.74 (0.18)	0.72 (0.18)
**Psychosocial super dimension**	0.41 (0.18)	0.43 (0.20)	0.43 (0.20)	0.43 (0.20)	0.41 (0.18)	0.41 (0.19)	0.43 (0.19	0.42 (0.19)
**Psychosocial subdimensions**								
**Mental Health**	0.61 (0.13)	0.63 (0.13)	0.63 (0.13)	0.63 (0.13)	0.61 (0.13)	0.61 (0.13)	0.63 (0.13)	0.63 (0.13)
**Happiness**	0.78 (0.14)	0.78 (0.14)	0.78 (0.14)	0.76 (0.15)	0.78 (0.15)	0.77 (0.14)	0.78 (0.14)	0.77 (0.15)
**Self-Worth**	0.86 (0.12)	0.87 (0.13)	0.86 (0.13)	0.86 (0.13)	0.86 (0.13)	0.85 (0.13)	0.87 (0.12)	0.85 (0.13)
**Coping**	0.77 (0.15)	0.77 (0.15)	0.77 (0.15)	0.77 (0.15)	0.77 (0.15)	0.76 (0.15)	0.78 (0.14)	0.78 (0.14)
**Relationships**	0.76 (0.16)	0.76 (0.17)	0.76 (0.17)	0.77 (0.17)	0.76 (0.15)	0.75 (0.16)	0.76 (0.16)	0.75 (0.16)
**Physical super dimension**	0.70 (0.21)	0.71 (0.20)	0.69 (0.21)	0.68 (0.21)	0.69 (0.19)	0.69 (0.20)	0.70 (0.20)	0.68 (0.21)
**Physical subdimensions**								
**Independent Living**	0.88 (0.14)	0.89 (0.14)	0.88 (0.15)	0.86 (0.15)	0.88 (0.13)	0.89 (0.14)	0.88 (0.14)	0.86 (0.15)
**Senses**	0.91 (0.08)	0.90 (0.08)	0.90 (0.08)	0.90 (0.09)	0.90 (0.09)	0.89 (0.09)	0.90 (0.09)	0.90 (0.09)
**Pain**	0.72 (0.26)	0.74 (0.25)	0.71 (0.24)	0.71 (0.25)	0.72 (0.23)	0.71 (0.25)	0.73 (0.24)	0.70 (0.25)

*Note: *All values are mean (standard deviation)*

© 2024 Hill A-M et al. *JAMA Network Open*

eTable 6. Secondary Outcomes Summary Statistics: Preparedness for Care (PCS), Caregiver Inventory (CGI), Family Appraisal of Caregiving Questionnaire (FACQ)

		**FECH+ Intervention; n=274**				**Control; n=273**			
	Time Point	**Baseline**	**3 Months**	**6 Months**	**12Months**	**Baseline**	**3 Months**	**6 Months**	**12 Months**
**Preparedness for Care**	Obs	n=274	n=237	n=226	n=174	n=273	n=240	n=218	n=177
	Mean	2.56	2.83	2.86	2.87	2.45	2.67	2.70	2.69
	SD	0.73	0.66	0.68	0.68	0.78	0.72	0.77	0.71
**Caregiver inventory**	Obs	n=274	n/a*	n=225	n=174	n=273	n=n/a*	n=218	n=177
	Mean	144.68	n/a*	149.02	152.14	144.58	n=n/a*	145.93	147.50
	SD	23.20	n/a*	24.66	22.46	24.09	n=n/a*	26.11	24.56
**Family Appraisal of Caregiving Questionnaire, Part 3**	Obs	n=274	n=237	n=226	n=174	n=273	n=240	n=218	n=177
	Mean	26.07	26.89	27.39	27.28	26.49	26.98	27.42	26.86
	SD	6.69	7.51	7.61	6.92	6.68	6.87	6.82	7.23
**Family Appraisal of Caregiving Questionnaire, Part 3**	Obs	n=274	n=237	n=226	n=174	n=273	n=240	n=218	n=177
	Mean	16.16	16.70	16.85	16.38	15.86	16.11	16.18	16.10
	SD	3.34	3.41	3.28	3.28	3.24	3.12	3.12	3.28

*Note: *n/a= time point at baseline, 6 and 12 months only. Obs=Observations; SD=Standard Deviation*

© 2024 Hill A-M et al. *JAMA Network Open*
