# Supplementary material for: Nurse Telephone Support for Caregivers of Older Adults at Hospital Discharge: A Randomized Clinical Trial
Source: JAMA Netw Open. 2024 Oct 25;7(10):e2441019. doi: 10.1001/jamanetworkopen.2024.41019 (PMC11581515; doi:10.1001/jamanetworkopen.2024.41019)
Supplement: Supplement 3. — Data Sharing Statement [file jamanetwopen-e2441019-s003.pdf]

Data Sharing Statement

Hill. Nurse Telephone Support for Caregivers of Older Adults at Hospital Discharge. *JAMA Netw Open*. Published October 25, 2024. doi:10.1001/jamanetworkopen.2024.41019

Data
Additional Information: ACTRN12620000060943 https://www.anzctr.org.au/
Data available: No

Additional Information
Explanation for why data not available: secondary analyses ongoing
